# Predicting West Nile virus transmission in North American bird communities using phylogenetic mixed effects models and eBird citizen science data

**DOI:** 10.1186/s13071-019-3656-8

**Published:** 2019-08-08

**Authors:** Morgan P. Kain, Benjamin M. Bolker

**Affiliations:** 1 Department of Biology, McMaster University, 1280 Main Street West, Hamilton, ON L8S 4K1 Canada; 2 Department of Mathematics and Statistics, McMaster University, 1280 Main Street West, Hamilton, ON L8S 4K1 Canada

**Keywords:** American robin, Dilution effect, Flavivirus, Multiple imputation, Phylogenetic analysis, Zoonotic spillover

## Abstract

**Background:**

West Nile virus (WNV) is a mosquito-transmitted disease of birds that has caused bird population declines and can spill over into human populations. Previous research has identified bird species that infect a large fraction of the total pool of infected mosquitoes and correlate with human infection risk; however, these analyses cover small spatial regions and cannot be used to predict transmission in bird communities in which these species are rare or absent. Here we present a mechanistic model for WNV transmission that predicts WNV spread (R_0_) in any bird community in North America by scaling up from the physiological responses of individual birds to transmission at the level of the community. We predict unmeasured bird species’ responses to infection using phylogenetic imputation, based on these species’ phylogenetic relationships with bird species with measured responses.

**Results:**

We focused our analysis on Texas, USA, because it is among the states with the highest total incidence of WNV in humans and is well sampled by birders in the eBird database. Spatio-temporal patterns: WNV transmission is primarily driven by temperature variation across time and space, and secondarily by bird community composition. In Texas, we predicted WNV R_0_ to be highest in the spring and fall when temperatures maximize the product of mosquito transmission and survival probabilities. In the most favorable months for WNV transmission (April, May, September and October), we predicted R_0_ to be highest in the “Piney Woods” and “Oak Woods & Prairies” ecoregions of Texas, and lowest in the “High Plains” and “South Texas Brush County” ecoregions. Dilution effect: More abundant bird species are more competent hosts for WNV, and predicted WNV R_0_ decreases with increasing species richness. Keystone species: We predicted that northern cardinals (*Cardinalis cardinalis*) are the most important hosts for amplifying WNV and that mourning doves (*Zenaida macroura*) are the most important sinks of infection across Texas.

**Conclusions:**

Despite some data limitations, we demonstrate the power of phylogenetic imputation in predicting disease transmission in heterogeneous host communities. Our mechanistic modeling framework shows promise both for assisting future analyses on transmission and spillover in heterogeneous multispecies pathogen systems and for improving model transparency by clarifying assumptions, choices and shortcomings in complex ecological analyses.

**Electronic supplementary material:**

The online version of this article (10.1186/s13071-019-3656-8) contains supplementary material, which is available to authorized users.

## Background

West Nile virus (WNV), a mosquito-borne pathogen of birds, is a model system for studying vector-borne disease transmission and virulence evolution [1–6]. West Nile virus caused infrequent outbreaks in Israel, Egypt, India, France and South Africa from 1937, when it was first isolated in Uganda, until the 1980s [4]. By the mid 1990s WNV had spread across much of Europe; it remains a moderate human and equine health burden in Europe and Africa today [5, 7–13]. West Nile virus was first detected in North America in New York, USA in 1999, and by 2003 had spread to all contiguous US states, southern Canada and northern Mexico [1], and has now become the world’s most widespread arbovirus [13]. The North American WNV epidemic caused population declines in numerous bird species [1, 14, 15] and hundreds of thousands of spillover infections in humans [16–18], including 23,000 reported cases of neuroinvasive WNV disease and more than 2000 deaths between 1999 and 2017 [19].

The life-cycle of WNV is sensitive to abiotic and biotic factors at every stage [4]. First, an infected mosquito infects susceptible birds (“mosquito-to-bird transmission”). Transmission probability during a feeding event depends on the viral load (titer) in a mosquitoes’ salivary glands, which is determined by the length of time the mosquito has been infected and the viral replication rate in the mosquito [20]; replication rate is a function of the dose the mosquito received when it became infected, the mosquito species and environmental variables such as temperature [6]. A mosquito’s overall ability to transmit infection to a susceptible host is called “vector competence” [21, 22]. Which bird species become infected depends on mosquitoes’ biting preferences [23] and on the abundance of each bird species in the community.

In the second step of transmission, infected birds infect susceptible mosquitoes (“bird-to-mosquito transmission”). The probability that a susceptible mosquito becomes infected during a feeding event depends on the titer in the bird species, the species of the mosquito and environmental variables such as temperature [6]. Critically, bird species vary considerably in both their physiological capacity for transmitting infection to mosquito vectors because of differences among species in survival and virus titer (which together comprise “host competence”), and in their relative contribution to the pool of infectious mosquitoes because of differences in their abundance and attractiveness to mosquitoes [23].

WNV has been intensely studied, including models and/or empirical analysis of prevention strategies for WNV [24, 25], ecological factors associated with the spread of WNV [26–29], risk assessment for invasion into new locations [11, 30–32], human infection risk [16, 33–36] and the importance of individual bird species in transmitting WNV [1, 6, 37, 38]. This work has contributed substantially to our understanding of the dynamics of WNV. For example, Wonham et al. [24] and four others reviewed in Wonham et al. [39] laid the foundation for WNV transmission models, providing insight into the threshold number of mosquitoes at which WNV R_0_ = 1 [24], the impact of bird mortality on transmission [40] and the transition from an epidemic to endemic state [41]. However, all of these studies used a differential equation framework that ignores much of the heterogeneity in transmission probabilities over the course of infection and variation among hosts and mosquitoes. Vogels et al. [29] do incorporate transmission probabilities from three vector species at three different temperatures; however, they considered only a single bird species. Kilpatrick et al. [1] and Peterson et al. [37] began to address the abundant variation in competence among bird species, which led to a variety of work on the connection between specific bird species and human infection risk [16, 35, 36].

Most work neglects much of the heterogeneity in the life-cycle of WNV: all of these analyses were focused narrowly on a small subset of the species found in diverse bird communities and/or use a small fraction of the available empirical data. Ideally, predictions for the spread of WNV in diverse communities of birds would be obtained from a mechanistic model that uses as much of the available empirical data as possible on individual-level processes to scale up to transmission at the level of the community while retaining the heterogeneities in WNV transmission. These data include, among many other axes of heterogeneity, the physiological responses of all of the bird species in the community, the biting preferences of mosquitoes on these bird species and the relative abundance of each bird species. Relative to phenomenological models, mechanistic models are often more powerful because they are better at prediction in conditions beyond those observed [42, 43], and help elucidate biological unknowns when they fail [44]. A mechanistic model for WNV would allow for estimation of the force of infection of WNV in any bird community and help researchers explore causal links between bird community composition and human infection risk.

In North America, there have been over 100 infection experiments of mosquitoes and birds (see [6] for a synthesis of these data), and extensive studies on mosquito feeding preferences (for a review see [23]; for examples of field observations see [45, 46]). Despite this work, bird communities across North America contain hundreds of bird species with unmeasured physiological responses to WNV and unknown mosquito biting preferences. Because of this gap, WNV spread has not yet been predicted mechanistically using full bird communities.

We present a model for predicting WNV R_0_ for bird communities in any state or province in North America (aggregated in space and time by county, month and year) or larger region; for details about R code see Additional file 1. While our model is set up to provide estimates of WNV transmission anywhere in North America, sufficient information about bird species abundance may be unavailable in some rural locations in the USA and many locations in Canada and Mexico. To get around the problem of unmeasured responses to WNV for many bird species, we estimated missing bird species’ responses using these species’ phylogenetic relationships with bird species with measured responses, a technique we call “phylogenetic imputation”. This is a general method that can be used to model the correlated responses of multiple species and efficiently estimate the response (e.g. traits, response to infection) of species with little or no data (see [47, 48] for a similar method and application). This technique allows us to scale up from the physiological responses of individual birds to disease transmission at the scale of the whole community by considering species-level variation in the physiological response to WNV and the biting preference by mosquito vectors of all of the birds in the community. This allows our model to retain all known heterogeneities in the life-cycle of WNV associated with the bird community.

A model allowing for all important WNV transmission heterogeneities would certainly need to allow for spatial and temporal variation in mosquito populations, temperature and the effects of temperature on transmission probabilities, mosquito survival and biting rate [12], each of which has a large effect on WNV transmission [29]. While our model considers spatial and temporal variation in temperature and resulting variation in transmission probabilities and mosquito survival, we assumed a single homogenous population of mosquitoes because of a lack of data on mosquito populations. Thus, while our model is a step in the right direction, ignoring variation in vector competence among mosquito species is a shortcoming of our approach.

We used a variety of datasets to fit our model including laboratory infections of birds and mosquitoes (full citations are available in Additional file 2; further details available in [6]), field data on mosquito biting preferences [45], bird body size data from a searchable database [49], bird detectability from field sampling (citations are listed in Additional file 2), the comprehensive phylogeny of birds [50, 51] and citizen science data on bird abundance from eBird, the Cornell Laboratory of Ornithology citizen science database [52].

We show how our model can be used to predict the intrinsic reproductive number (R_0_) of WNV, the expected number of new infections a single infected individual generates in an otherwise susceptible population. We focused our analysis on Texas, USA, because it is among the states with the highest total incidence of WNV in humans [53] (Texas had an estimated total of 534,000 cases between 2003 and 2010 [16], and Dallas county specifically had the highest recorded number of cases anywhere in the USA in a 2012 nationwide WNV epidemic [54]), and is well sampled in the eBird database. We used R_0_ as a metric to compare transmission potential among bird communities; we did not use R_0_ as a metric to predict the exact size of a new epidemic, which would require detailed information on bird seroprevalence. We examined spatio-temporal patterns in WNV R_0_ across Texas and determined which bird species in Texas are the best and worst hosts for propagating WNV. For this case study we assumed a single mosquito species, which allows us to address our primary focus of variation in the bird community.

Using our imputed responses for full bird communities and R_0_ estimates in Texas, we tested both an assumption and a prediction of the dilution effect hypothesis, which argues that increasing biodiversity (in either species richness or evenness) will decrease R_0_ or another quantity associated with the spread of disease such as the number of spillover infections into non-target hosts [55–57]. Previous work in this system has found variable support for the dilution effect hypothesis [28, 33, 58, 59]. In an attempt to clarify these variable results, we tested if more abundant bird species are better hosts for WNV (an assumption of the dilution effect hypothesis), and whether bird species richness is positively or negatively correlated with WNV R_0_ (an amplification or dilution effect respectively).

We structured our paper and supplemental code to serve as a reference for future work analyzing ecological problems that require multi-faceted mechanistic models, which consist of many sub-models that may use different data sources. We provide a detailed description of each of our sub-models and give reasons for our statistical choices; we emphasize principled ways to estimate missing data, and the importance of propagating uncertainty. Additional file 1 provides details on how to access extensively commented R code and a complete list of all data cleaning and analysis steps required to obtain estimates for the R_0_ of WNV in any region in North America using a single compressed eBird data file available upon request from [60].

## Methods

### Model overview

We introduce our model by working backwards, from the overarching biological questions to the specifics of individual models. We begin by describing our primary model outcomes. We then explain our method for calculating the R_0_ of WNV. Finally, we detail how we estimated each parameter in the equation for R_0_ using individual sub-models, and how we linked these estimates and propagated uncertainty to calculate R_0_. Table 1 describes the components of our overall model and how they fit into our analysis.

**Table 1:** Sub-model details for our multi-faceted ecological model for WNV R_0_. The two transmission steps (Column 2) of WNV’s life-cycle are: mosquito to bird (M-to-B) transmission, i.e. transmission from an infected mosquito to a susceptible bird; and bird to mosquito (B-to-M) transmission, i.e. transmission from an infected bird to a susceptible mosquito. Citations accompany data available in Additional file 2; details on data extraction can be found in [6]

Component of community R_0_	Transmission step	R_0_ equation component (see Eq. 2)	Data source	Details in
Raw eBird counts of bird species *i*	M-to-B	Component of ω_Si_ and ω_µi_	1,437,050 complete lists submitted between 2000 and 2017 in Texas, USA	Methods, Model components: Bird community
Detectability of bird species *i*	M-to-B	Component of ω_Si_	12 publications including estimates for 475 bird species	Methods, Model components: Bird detectability
Mosquito biting preference on bird species *i*	Both	Component of ω_Si_	[45] and eBird records for the same spatio-temporal sampling period	Methods, Model components: Mosquito biting preference
Mosquito incubation of WNV	M-to-B	Determines P_MBd_	9 publications including 45 infection experiments (see Additional file 2 and [6])	Methods, Community R_**0**_; model from [6]
Mosquito survival	M-to-B	S_Md_	[128]	Methods, Community R_**0**_; model from [6]
Mosquito biting rate	Both	δ	[46] from [24] and [62]	Methods, Community R_**0**_
Titer profile of bird species *i*	B-to-M	T_ij_	30 publications including 111 infection experiments of 47 bird species (see Additional file 2 and [6])	Methods, Model components: Bird titer profile and survival
Survival of bird species *i*	B-to-M	S_Bij_	30 publications including 111 infection experiments of 47 bird species (see Additional file 2 and [6])	Methods, Model components: Bird titer profile and survival
Bird-to-mosquito transmission probability	B-to-M	P_BMij_	20 publications (see Additional file 2 and [6])	Methods, Model components: Bird titer profile and survival; model from [6]
No. of mosquitoes per bird	B-to-M	n_MB_	Based loosely on [46]	Methods, Community R_**0**_

### Model outcomes

First, we focused on spatial and temporal patterns in R_0_ at the level of the community; we calculated WNV R_0_ for bird communities between 2000 and 2017 separated spatially by county and temporally by month and year, and then fit a spatio-temporal model to the resulting WNV R_0_ estimates which included 11 ecoregions in Texas, human population density, temperature and year as predictor variables. Secondly, we determined which bird species have the largest predicted impact on R_0_ in Texas, USA. We quantified the importance of each species within each community by calculating the proportional change in R_0_ that would be predicted to occur if that species were removed from the community and replaced by the other species in community in proportion to their relative abundance. We considered species whose removal strongly increases or decreases R_0_ as the least or most competent birds for WNV, respectively. In the language of the dilution effect hypothesis [55–57], species that increase R_0_ when removed are defined as “diluters”, and those that decrease R_0_ when removed as “amplifiers”. We test if more abundant bird species are more physiologically competent for transmitting WNV and if an increase in species richness is predicted to decrease WNV R_0_.

### Community R_0_

We calculated R_0_ as the expected number of mosquitoes that become infected following the introduction of a single infected mosquito into a population of susceptible birds and otherwise uninfected mosquitoes. This calculation assumes that all mosquitoes have identical biting preferences and vector competence. We broke R_0_ into two transmission steps: mosquito-to-bird transmission, which measures the expected number of each bird of species *i* that would become infected by a single infected mosquito; and bird-to-mosquito transmission, which calculates the expected number of mosquitoes infected by the infected birds of species *i* calculated in the mosquito-to-bird transmission step. Written in this way, the sum of bird-to-mosquito transmission gives the number of new infected mosquitoes resulting from the single infected mosquito, which is the R_0_ of WNV.

Mosquito-to-bird transmission is calculated by:

1 $$\mu_{i} = \omega_{Si} \mathop \sum \limits_{d = 1}^{D} P_{MBd} S_{Md} \delta ,$$

where *µ*_*i*_ is the number of birds of species *i* that become infected when a single infected mosquito is introduced into a community of susceptible birds. The quantity *ω*_*Si*_ is the scaled proportion of susceptible individuals of bird species *i*, which is given by the observed proportions of species *i* (determined by eBird data; see “Methods”, Bird community), weighted by the detectability of species *i* (see “Methods”, Bird detectability) and the mosquito biting preference on species *i* (see “Methods”, Mosquito biting preference). The derivation of *ω*_*Si*_ is given in Methods, Mosquito biting preference. Total transmission from the infected mosquito to susceptible birds is given by a sum over *D*, the duration of the mosquito’s infectious period. This sum is a measure of vector competence, the total ability of a vector to transmit infection to a susceptible host [21], a key component of which is the transmission probability per feeding event [22]. The probability of transmission per mosquito bite on each day (*P*_*MBd*_) follows a logistic function of titer in the mosquito’s salivary glands, which is a function of time since infection, dose received from the infected bird, temperature, mosquito species and WNV strain (suppressed for clarity in Eq. 1; see [6] for a synthesis of these data). Here we assumed that the mosquito is introduced into the susceptible population of birds on the first day following infection with the WN02 strain of WNV with a dose of 10^5.5^ viral particles. We predicted mosquito incubation rate of WNV and mosquito survival (*S*_*Md*_; estimates for mosquito survival are taken from a model for mosquito survival fit in [6]) for each Texas bird community using the average temperature in each Texas county by month and year with temperature data obtained from NOAA [61]. We ignored the effect of mosquito species, which was fitted as a random effect in [6], due to the absence of data. These simplifications do not affect the relative effect of bird species, but will affect overall R_0_ values, and could affect spatio-temporal patterns. Finally, *δ* is mosquito biting rate with units of bites per mosquito per day. We assumed a constant mosquito biting rate of 0.14 per mosquito per day (as assumed by [46], taken from [24, 62]).

WNV R_0_ is calculated using the sum of bird-to-mosquito transmission:

2 $${\text{R}}_{0} = \sum\limits_{i = 1}^{I} {\left( {\omega_{\mu i} \mathop \sum \limits_{j = 1}^{8} \left( {P_{BMij} \left( {T_{ij} } \right) \cdot S_{Bij} } \right) n_{MB} \delta } \right)} ,$$

Equation 2 gives the expected number of new mosquitoes infected by the expected number of birds of species *i* that became infected by the single infected mosquito in mosquito-to-bird transmission (given by *µ*_*i*_ in Eq. 1), the sum of which is the R_0_ of WNV. In Eq. 2, $\omega_{\mu i}$ is *µ*_*i*_ from Eq. 1 weighted by the biting preference of mosquitoes on each species. The transmission probability from an infected bird of species *i* to a susceptible mosquito on day *j* (*P*_*BMij*_) is a function of a bird’s titer (*T*_*ij*_). Transmission probability is discounted by the bird’s survival probability up to day *j* (*S*_*Bij*_). We measured bird titer and survival until day 8, which is one day longer than previous measures of host competence [20, 63] and long enough to capture all known detectable measures of titer in birds. The inner summation over *j* captures a quantity commonly called “host competence”, which we call “physiological competence” to emphasize that this component is not scaled by mosquito biting preference. Classically, host competence is defined as the daily sum of host-to-vector infection probability over the course of a host’s infectious period [20, 63], assuming a single mosquito bite per day on an infected bird. Here, when multiplied by *ω*_*µi*_, this quantity gives the number of new mosquitoes that infected individuals of species *i* infect, arising from a single originally infected mosquito (in Eq. 2 the entire quantity inside the outer parentheses). The R_0_ of WNV is given by the sum of this quantity over all infected bird species multiplied by a constant ratio of mosquitoes to birds (*n*_*MB*_) (in the absence of better data we assume a ratio of 3 based approximately on sampling conducted by [46] in New Haven, CT, USA) and the number of bites per mosquito per day (*δ*).

We focused on estimating the parameters associated with the bird community, which include *ω*_*Si*_, *ω*_*µi*_, *P*_*BMij*_, *T*_*ij*_ and *S*_*Bij*_. For mosquito-to-bird transmission probability (parameters *P*_*MBd*_ and *S*_*Md*_) we used estimates from the models fit in [6] and single values from the literature for mosquito biting rate (*δ*) and the ratio of mosquitoes to birds (*n*_*MB*_). While the mosquito-to-bird ratio and mosquito biting rate will in reality be a function of parameters that vary both spatially and temporally such as ecoregion, season and temperature [29], as well as human population density, we assumed a constant mosquito-to-bird ratio here because of a lack of sufficient data on spatial and seasonal variation in this ratio across Texas and because our primary focus is on estimating R_0_ as a function of the bird community. Because we assumed no interaction between mosquito species and bird species in the probability of infection, and because the remaining parameters are scalars, differences in these parameters will affect the overall magnitude of R_0_ estimates but will not affect qualitative patterns in R_0_ due to variation among bird communities in space and time.

In Methods, Model components, we further unpack Eqs. 1 and 2 (e.g. *ω*_*Si*_) and describe how we estimated each of the parameters associated with the bird community. The data and models that informed all parameters of both Eqs. 1 and 2 are described in greater detail in Table 1.

### Phylogenetic imputation

The primary difficulty in estimating community competence for a diverse community of birds is that physiological responses to WNV and mosquito biting preferences are unknown for most bird species. Obtaining these data for every species in a diverse community of birds would be infeasible. To address this problem, we used a form of phylogenetic analysis that we call “phylogenetic imputation” in which we fit models using all of the data that are currently available for a given response (e.g. a bird species’ titer profile) and estimate the response of species with missing data using the phylogenetic relationship between the missing species and the species for which we have data [47].

The effects of a predictor variable on the response of multiple species can be modeled using the phylogenetic relationships among the species to estimate the correlation among observations. Classic phylogenetic regression approaches assume a correlated-residual model using phylogenetically independent contrasts (PICs), where the residuals evolve as a Brownian motion process [64]; in other words, residuals are phylogenetically correlated. Many recent approaches, including phylogenetic generalized linear mixed models (PGLMM) [65], Pagel’s *λ* [66] and Blomberg’s *κ* [67], expand upon Felsenstein’s PICs by incorporating extra parameters that correct for bias, and by partitioning the phylogenetically correlated residual variation into phylogenetically uncorrelated residual variation (observation error or tip variation) and phylogenetic signal (biological/evolutionary process error) [68].

Here we used a newly implemented method built on the *lme4* package in R that incorporates phylogenetic correlations by modeling them as random effects and allows for random slopes (i.e. phylogenetic signal in response to change in the predictor variable), random interactions and nested random effect models, and is orders of magnitude faster than alternative methods [69]. Like most previous methods, the evolutionary history for each species is modeled as a sequence of normal independent errors. Thus, the portion of a species’ response attributable to its evolutionary history can be calculated as the sum of the evolutionary change that occurred on each of the internal branches in the phylogeny leading to that species.

We estimated missing values for bird responses (e.g. bird titer) using multiple imputation (where each missing value is replaced by random samples from a distribution of plausible values [70]). To impute, we first fitted a phylogenetic mixed model to all of the species for which we have data. Then, for each species without data, we first summed the evolutionary change in the response variable that occurred on all branches of the phylogeny leading to the most recent common ancestor between the species with a missing response and the most similarly related species that has data and was included in the mixed model. This gives the effect on the response variable of the species’ shared evolutionary history up to the time when these species diverged. To obtain these values we drew random normal (multivariate if the mixed model includes multiple correlated species-level random effects) samples for each branch, with means equal to the conditional modes of each branch multiplied by the branch length and variances equal to the conditional variances of each branch multiplied by the square of the branch lengths. Then, the evolutionary change that has occurred since the two species diverged is estimated by drawing random normal (multivariate normal if the mixed model includes multiple correlated species-level random effects) samples with a mean of zero (the expected value for each unmeasured species is equal to that of the most closely related measured species because of the assumption of Brownian motion) and standard deviation (SD) equal to the estimated SD of the species-level random effect(s) multiplied by the evolutionary distance (branch length) from the most recent common ancestor of the most closely related measured species. Together, these estimates give the estimated total effect of a species’ evolutionary history on a given response. The remaining portion of a species’ response is given by the fixed effects (e.g. body size) and other non-species-level random effects (e.g. variation among infection experiments).

For our analysis we used a bird consensus phylogeny that was calculated using 1000 trees downloaded from [50] (Stage2_MayrPar_Ericson_set1_decisive.tre) [51] using DendroPy [71] and methods described in [72].

### Phylogenetic imputation validation

We validated our phylogenetic imputation method in two ways. First, we calculated conditional *R*^2^ using the methods outlined in [73, 74] for models with and without a species-level phylogenetic random effect. We estimate conditional *R*^2^ using code from the R package *MuMIn* [75], adapted to accommodate the structure of the phylogenetic mixed model objects. Secondly, we use blocked leave-one-out cross validation [76] at the level of species for models with and without the species-level phylogenetic random effect to assess the effects of phylogenetic imputation on out-of-sample error. We present additional details and results for each of these forms of validation in Additional file 2: Text S2; Table S1. For a vignette on the phylogenetic models built on *lme4* see [69].

### Model components

#### Bird titer profile and survival

We modeled bird infection profiles and mortality probabilities using data from experimental infections of 47 bird species collected from 30 publications containing 113 individual infection experiments; most of these data have been presented previously [6]. For the bird titer, bird survival and bird-to-mosquito transmission models in this paper we grouped data from the two primary WNV strains, NY99 and WN02 (in our previous study [6] we were unable to detect a clear difference between the NY99 and WN02 strains).

To model bird titer profiles we used a log-normal mixed effects model; fixed effects included a Ricker function of day (using day and log(day) as predictors of log-titer; see Additional file 2: Text S3 or [77] for more information), infectious dose, bird body size and the interaction between day and bird body size. We used a random intercept and slope over both day and log(day), which are constrained by the phylogenetic relationship among the species. We also included random intercepts for citation and infection experiment.

To model bird survival we used a generalized linear mixed effects model (GLMM) with a binomial error distribution and complementary log-log link, where the number of birds dying on a given day was taken as the number of “successes” and the number of birds that survived that day as “failures”. This model estimates a bird’s daily log-hazard [78], which can be back-transformed to estimate daily mortality probability and cumulative survival probability using the cumulative product of the complement of the daily mortality probabilities. We modeled bird survival using the main effects of titer, day and bird body size as fixed effects; citation, infection experiment and bird species (phylogenetically constrained) were modeled using random intercepts (due to a lack of data we were unable to estimate species-level variation in sensitivity to titer).

The bird body size data used in both models were obtained from the searchable digital edition of Dunning [49]. Body size data were averaged if data for a given species were available for both sexes or multiple subspecies. Approximately 7% of the species in the Texas eBird dataset did not have mean body sizes reported in [49] but did have minimum and maximum values reported. The body size for these species was taken as the center of the range. Approximately 0.3% of the species in the Texas eBird dataset were not represented at all in [49]. For these species, the body sizes of all congeners were averaged.

#### Bird community

We obtained bird abundances data from the Cornell Laboratory of Ornithology citizen science database eBird [52, 60]. We used all complete checklists [52, 79] submitted between January 2000 and December 2017. Complete checklists are defined as a report of all birds (number of individuals of all species) that are seen on a given outing. Checklists were aggregated spatially at the level of Texas counties for each month between January 2000 and December 2017, which resulted in a total of 30,188 bird communities containing a total of 679 unique species. To match scientific names, which occasionally differed between eBird and the consensus phylogeny, we used an automated lookup procedure to search both the IUCN [80] and Catalogue of Life [81] databases. All unmatched names following the automated lookup were matched by hand using manual searches (*<* 1% of species).

We focused on results for a reduced eBird dataset that included 2569 communities and a total of 645 bird species, with a median occurrence (proportion of communities in which bird species *i* was sampled) of 13% (95% of species between 0.04% and 82%; a total of 167 species were recorded in less than 1% of the communities). We present results for the complete Texas eBird dataset, which included all 30,188 available communities and 679 species in Additional file 2: Figures S4, S5; Text S5. We subset our data for the main analysis because many of the Texas bird communities were under-sampled (e.g. 13,254 communities were sampled with 5 or fewer lists) and therefore these data are unlikely to be a good representation of the true bird community. The 2569 bird communities were chosen because they were all sampled with a minimum effort of 80 complete checklists. We chose 80 lists in an attempt to maximize the number of communities for our analysis while minimizing the retention of under-sampled communities. To optimize the tradeoff between number of communities and data quality, we resampled 5–120 complete lists from the 46 most sampled communities (communities with greater than 1300 lists) 100 times. We calculated the proportion of species missing in the subsampled communities as well as the root mean squared error (RMSE) in the relative proportions of all species between the two communities. Using the rate of change in RMSE and species retention (Additional file 2: Figures S1, S2), we determined that with fewer than 80 complete lists, the gain in total number of communities was not worth the increased error rate and loss of species representation, while at greater than 80 complete lists the loss in communities was too large for the small decrease in error and species loss. For full simulation results see Additional file 2: Text S1; Figures S1, S2).

#### Bird detectability

We scaled raw bird counts by the detectability of each bird species to correct for incomplete sampling of bird communities and to control for variation in the quality of eBird records; alternatively or additionally, eBird lists can be weighted by user skill [79, 82]. We searched for data on the maximum detection distances of birds using Google Scholar with the following search criteria: “X” maximum detection distance, “X” maximum detection radius, “X” effective detection distance and “X” effective detection radius, where “X” took each of: landbird, land bird, waterbird, water bird, waterfowl, seabird, sea bird, and marsh bird. In all cases the first 60 hits were assessed for relevant information. Because of overlapping results, a total of 1440 titles, abstracts and/or entire papers were read for relevant data. In total, we took data from 12 sources which contained maximum detection distances for 469 bird species. However, we failed to find detection distances for waterfowl and shore birds; maximum detection distances (roughly intermediate to values between woodland species and seabirds) were assigned to 21 waterfowl and shore birds based on detection probabilities in the literature and our knowledge of the natural history of these species (personal birding experience [83]).

In order to fill in missing information for detection distance, we used the results of our literature search to fit a phylogenetic mixed effects model. Maximum detection distances for species in the Texas eBird data were estimated using a GLMM with a log-normal error distribution. Body size was used as a fixed effect and species was included as a phylogenetic random effect. The eBird counts for each species were then adjusted by multiplying counts by the ratio of the maximum detection distance in the community to the detection distance of each species. Using the square of maximum detection distance to reflect the relative spatial area sampled for each species may also be an appropriate method for adjusting raw eBird counts. We chose linear scaling here because 50% of lists were transects and because squared distance generated unrealistic outliers.

#### Mosquito biting preference

Finally, bird species proportions were adjusted using the biting preferences of mosquitoes, which scales true bird proportions to the proportions that mosquitoes “see”. Because mosquitoes (*Culex* sp. and others) prefer some hosts to others [20, 45, 84], this step is required to appropriately translate each bird’s physiological response (a bird’s mosquito infecting potential) into realized infections of mosquitoes [20]. A mosquito’s biting preference on bird species *i* can be calculated as the rate of mosquito feeding on species *i* relative to its abundance in the community [20]: $$\beta_{i} = {\raise0.7ex\hbox{${f_{i} }$} \!\mathord{\left/ {\vphantom {{f_{i} } {a_{i} }}}\right.\kern-0pt} \!\lower0.7ex\hbox{${a_{i} }$}}$$, where *f*_*i*_ is the fraction of total blood meals from species *i*, and *a*_*i*_ is the proportion of species *i* in the community. Experimentally, *f*_*i*_ is determined by sampling mosquitoes and determining the species origin of blood recovered from the mosquitoes; bird surveys are used to determine *a*_*i*_ [20, 45, 46]. A value for *β*_*i*_ = 1 indicates that a bird species is bitten exactly in proportion to its representation in the community. A value of *β*_*i*_ *>* 1 or *β*_*i*_ *<* 1 indicates a bird species that is preferred or avoided by mosquitoes, respectively. At one extreme, a bird with high infectious potential (high titer and low mortality) may contribute very little to the spread of WNV if it is avoided by mosquitoes. At the other extreme, a bird with low physiological competence (low titer and/or high mortality) may contribute substantially to the spread of WNV if it is among the most preferred species in a community. For example, American robins (*Turdus migratorius*) have been found to infect the largest, or close to the largest, proportion of mosquitoes of any bird species in some bird communities in eastern USA because of their high abundance and mosquito preference [20, 45, 46, 85, 86], in spite of their relatively low titer [6].

In previous studies, when the blood of bird species *i* was recorded in a mosquito, but bird species *i* was unobserved in the community, the bird was either assigned a proportion corresponding to the rarest bird measured [45], or dropped from the analysis [20]. If bird species *i* was observed but its blood was not detected in a mosquito, it was assumed that a single mosquito was observed with the blood of bird species *i* [20, 45]. While convenient, the assignment of arbitrary values to missing data leads to biting preferences spanning three orders of magnitude [20, 45], which seems biologically implausible. Alternatively, a Bayesian statistical model can be used to estimate mosquito biting preference (which is not directly observed), when bird species *i* or its blood is not observed. Here we use a multinomial model in Stan [87], interfaced with R using *rstan* [88]. We model bird proportions using data from [45] and a Dirichlet prior, the conjugate prior to the multinomial distribution [89]. The Dirichlet prior was set proportional to eBird observations for the same location and dates as the sampling originally conducted in [45]; we used all complete checklists in a circle with radius 08’ around the focal point of 41°42′N, 87°44′W given as the center of the surveys conducted in [45] for the months of May and October in 2006–2008; this area is shown in Additional file 2: Figure S3.

The fraction of total blood meals in mosquitoes was modeled using a Gamma error distribution with data from [45] and a Gamma prior (shape = 0.25, scale = 0.25). This prior distribution has a mean equal to one, median less than one and moderate dispersion, which assumes that birds are preferred in proportion to their abundance on average; the majority of bird species are preferred a bit less than proportional to their relative abundance, while a few bird species are preferred much more than proportional to their relative abundance. This Dirichlet-multinomial model estimates mosquito biting preferences for all of the species recorded on eBird between May and October in 2006–2008 in Cook County, IL, USA.

Estimates of mosquito blood meals from the Dirichlet-multinomial Stan model were then used to impute biting preference on bird species in the Texas dataset by fitting a GLMM with Poisson-distributed error (which includes a species-level phylogenetic random effect) to the biting preferences estimated by the Dirichlet-multinomial model. This step assumes that a mosquito’s biting preference on species *i* is the same in Illinois as in Texas; both states share *Cx. tarsalis*, while *Cx. pipiens* is unique to Illinois and *Cx. quinquefasciatus* is unique to Texas [90], making this an unavoidable oversimplification. Biting preference estimates were scaled to a mean of one, and were then used to weight the observed proportions of each bird species. The weighted proportions of each bird species were obtained using:

3 $$\omega_{\iota } = \frac{{\beta_{i} \alpha_{i} \delta_{i} }}{{\mathop \sum \nolimits_{i = 1}^{I} \beta_{i} \alpha_{i} \delta_{i} }},$$

where *ω*_*i*_ is the adjusted proportion of species *i*, *α*_*i*_ is the unweighted proportion of each species determined directly from eBird data, *β*_*i*_ is mosquito biting preference on species *i*, and *δ*_*i*_ is the ratio of the maximum bird detectability in the community to the detectability of species *i*. The scaling in this equation is equivalent to using a weighted Manly’s *α* index [91].

#### Spatio-temporal patterns in WNV R_0_

To determine the spatio-temporal patterns in WNV R_0_ we fit a generalized additive model (GAM) using the *mgcv* package in R [92]. We use this as a proof of concept example to show how the imputed physiological responses of birds and mosquito biting preferences can be used to predict larger scale patterns. This model included thin plate splines for the log of human population density, temperature and year. We stress that in the absence of data on mosquito communities on the scale of the bird communities, the R_0_ estimates from this model are driven by variation in bird communities and temperature only and cannot be taken at face value as accurate estimates of actual WNV transmission potential.

We first attempted to fit a model using the proportion of each ecoregion in each county, but could not overcome issues of concurvity (analogous to co-linearity in a GAM model [93]) in this model. Instead, we fitted a simplified model using a Markov random field to model the effects of ecoregion under the simplified assumption that each county had only a single ecoregion, which we chose as the most abundant ecoregion in each county. We fitted a random effect of county to control for repeated measures within counties and to account for spatial variation within ecoregion. Ideally, we would also model fine-scale spatial variation using a thin plate spline over latitude and longitude coordinate pairs; however, models that included this predictor suffered greatly from concurvity problems. We used the inverse of the variance in R_0_ estimates as weights.

For the 11 major different ecoregions in Texas, population density and county spatial shape data were obtained from [94]. This model provides estimates of both seasonal and long-term trends in WNV R_0_ as the structure of bird communities have changed in the past two decades (due to disturbances such as habitat change [95], habitat destruction [96], climate change [97] and the effect of the WNV epidemic itself [1]) as well as spatial estimates of WNV R_0_ by county.

#### Propagation of uncertainty

Multi-faceted ecological models will underestimate uncertainty (e.g. too narrow confidence intervals on estimates of outcomes of interest) if the point estimates from each sub-model are used while neglecting their uncertainty. Point estimates may also differ between models with or without uncertainty because non-linear transformations of distributions will change the expected value, a phenomenon known as Jensen’s inequality [98]. We focus on results from a model with all uncertainty propagated, but briefly discuss the impacts of ignoring uncertainty on both our quantitative and qualitative conclusions (for more detailed results see Additional file 2: Figure S6). Table 2 gives a list of the sources of uncertainty and how each source was propagated. We set up our sub-models in the R code (see Additional file 1) so that each source of uncertainty can be set individually to be either propagated or ignored, which can be used to obtain a first approximation (assuming independence of errors) for the relative effects of uncertainty in each sub-model on uncertainty in R_0_ and on spatio-temporal patterns in R_0_. We briefly discuss which sources of uncertainty have the largest impact on our conclusions in Additional file 2: Single sources of uncertainty: Reduced eBird Dataset.

**Table 2:** Details about each source of uncertainty

Source of uncertainty	Description	Method of propagation
Fixed effects	Uncertainty in the fixed effects for each sub-model	1000 multivariate (or univariate depending on the model definition) normal samples using the means and vcov matrix of the fixed effects
Phylogenetic random effect	Uncertainty in the amount of evolutionary change in the response variable (e.g. bird titer) that has occurred over each branch of the phylogeny	1000 multivariate (or univariate for models with a single species-level random effect) normal samples for each branch, with means equal to the conditional modes of the species-level random effect for each branch multiplied by the branch lengths and variance equal to the variance of the conditional modes of the random effects for each branch multiplied by the squared branch lengths
Phylogenetic tip variation	Evolutionary change that has occurred after the divergence of the species whose response is being imputed from its most closely related species that has an empirically measured (and estimated) response	1000 multivariate (or univariate for models with a single species-level random effect) normal samples with mean 0 (because of the assumption of Brownian motion), and SD equal to the SD of the species-level random effect multiplied by the length of the final (most recent in time) branch leading to the species in question
Other random effects	Uncertainty due to variation among studies and infection experiments	1000 univariate normal samples for each random effect with mean equal to 0 and SD equal to the estimated SD
Stan model overall uncertainty	Summary of the entire uncertainty associated with the three Stan models used in the transmission steps between mosquitoes and birds (bird-to-mosquito transmission probability, mosquito-to-bird transmission probability, and mosquito biting preference)	1000 samples from the posterior distributions for each of the Stan models

*Abbreviation*: SD, standard deviation; vcov, variance-covariance

## Results

### Community R_0_

**Fig. 1:**
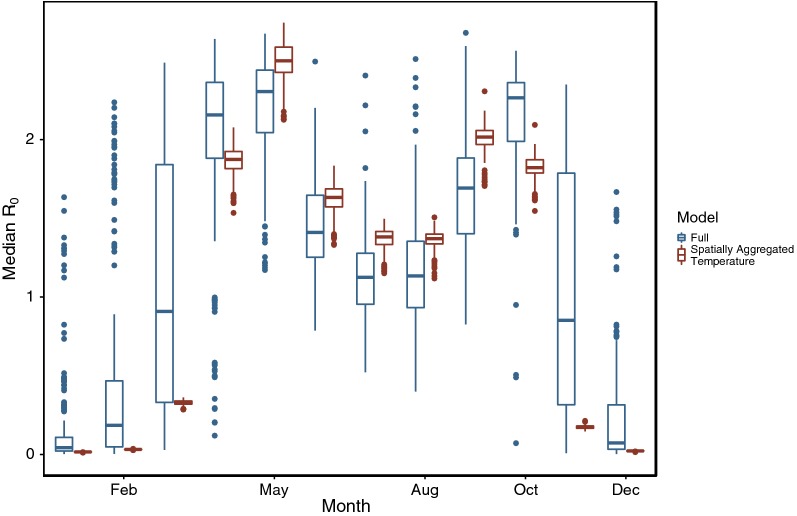
WNV R_0_ estimates between months and among Texas counties. Blue boxplots show R_0_ estimates across Texas counties within months for a “Full” model, which used the eBird community and NOAA temperature data for each community. Red boxplots show R_0_ estimates from a model where each community retained their specific eBird community, but whose temperature was replaced with the average temperature across all of Texas for that month (also see Table 3, Spatially averaged temperature). Variation in R_0_ within months attributable to variation in the bird communities (red boxplots) is considerably smaller than the variation explained by spatial variation in temperature. Increases or decreases in medians between the models within months is due to the effects of averaging temperature prior to predicting R_0_ using the non-linear functions for mosquito-to-bird transmission and mosquito survival across temperature, a manifestation of Jensen’s inequality. For example, in November the mean temperature across Texas is 13.6 °C, while the SD among counties is 3.30 °C. We estimate average mosquito-to-bird transmission per bite over the first 30 days of mosquito infection to be 2.5% at 13.6 °C, 8.5% at 16.9 °C (+ 1SD) and 25% at 20.2 °C (+ 2 SD)

WNV transmission is controlled primarily by temperature variation across time and space. In Texas, we estimated WNV R_0_ to be highest in the spring and fall when temperatures maximize the product of mosquito transmission and survival probabilities (across all ecoregions in April: median R_0_ = 2.16, median temperature across all Texas counties = 19 °C; May: R_0_ = 2.31, 23 °C; October: R_0_ = 2.27, 19 °C) (Fig. 1). Within these favorable months, we estimated R_0_ to be highest in the “Piney Woods” ecoregion (median R_0_ = 2.29) and “Oak Woods & Prairies” (median R_0_ = 2.28) ecoregions of Texas, and the lowest in the northern “High Plains” ecoregion (median R_0_ = 1.46). Despite these large differences at the larger scale of ecoregions, large uncertainty in the R_0_ of individual communities makes it difficult to be certain about the size of the true variation in space and time. For example, despite median estimates of R_0_ > 1 for 96% of communities in the most favorable months, the 95% CI for all of these communities includes R_0_ = 1 (the median across communities of the lower bound of the 95% CI of R_0_ is 0.60). In the least favorable months (e.g. December and January), 100% of community median R_0_ estimates were less than 1, while 67% of the CI for these communities spanned one (the median of the upper bound of the 95% CI is 2.0).

We decomposed the importance of spatial and temporal variation in both temperature and bird community composition by comparing the mean absolute deviation (MAD) in predictions for R_0_ between a full model and models with either the bird community or temperature aggregated across space or time (Table 3). Temperature variation across both space and time is more predictive of R_0_ than bird community composition, though ignoring variation in the bird community across space does lead to R_0_ estimates that differ from the full model by 0.17 on average (Table 3). Allowing for temporal variation in temperature and bird community composition, the majority of the variation in R_0_ within single months is due to spatial variation in temperature; variation in bird community composition is the next most important term (Fig. 1).

**Table 3:** Capability of simplified models to estimate WNV R_0_ in Texas. Mean absolute error compares R_0_ estimates from a simplified model to the R_0_ estimates from a full model for all 2569 of the bird communities in the reduced eBird dataset

Model	Mean absolute error in R_0_ estimates
Temporally averaged bird community^a^	0.07
Spatially averaged bird community^b^	0.15
Spatially averaged temperature^c^	0.36
Temporally averaged temperature^d^	0.40
Mean model^e^	0.63

^a^Temporally averaged bird community: each counties’ bird community is replaced with the average bird community in that county across all months

^b^Spatially averaged bird community: each counties’ bird community in each month is replaced with the average bird community across all of Texas in that month

^c^Spatially averaged temperature: each counties’ temperature in each month is replaced with the average temperature across all of Texas in that month

^d^Temporally averaged temperature: each counties’ temperature is replaced with the average temperature in that county across all months

^e^Mean model: each counties’ bird community and temperature is replaced with the average bird community and temperature across all counties and all months

**Fig. 2:**
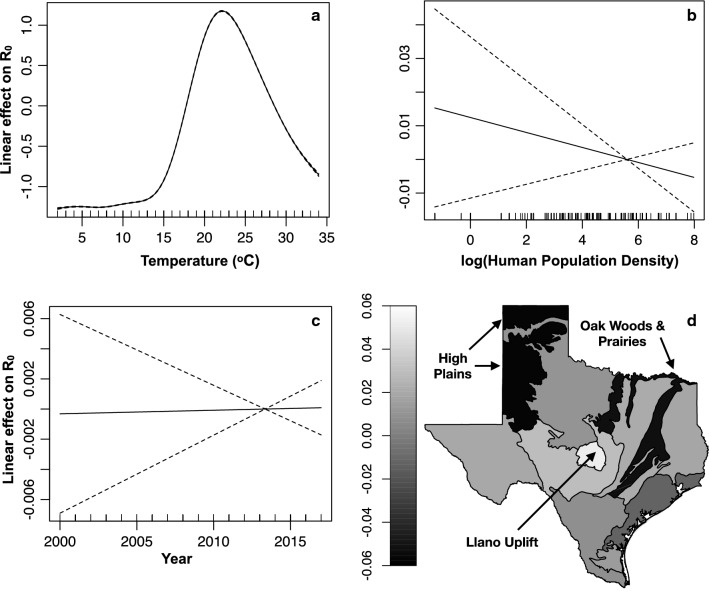
Spatio-temporal GAM model parameter estimates. Y-axes in panels **a**–**c** and the gradient in panel **d** show the additive effect of centered covariates on R_0_. The gradient in panel **d** shows variation in R_0_ among ecoregions explained by variation in bird communities. Dashed lines show 95% CI

The spatio-temporal GAM model explained 99% of the variation in estimated WNV R_0_; results for the spatio-temporal model are presented visually in Fig. 2. Most of the variation in WNV R_0_ is explained by temperature in the fitted GAM (Fig. 2a). Human population density (people/sq.mile) was associated with decreasing R_0_, but with small effect and large uncertainty (Fig. 2b). Ignoring the effects of fluctuations in mosquito populations, WNV R_0_ was estimated to vary little across years (Fig. 2c). Variation due to bird communities among ecoregions after controlling for temperature explained a small fraction of the variation in R_0_ among regions (Fig. 2d). Our fitted GAM predicts that bird communities in the “High Plains” and “Oak Woods & Prairies” ecoregions are the least favorable for WNV transmission, while bird communities in the “Llano Uplift” are the most favorable (Fig. 2d).

To evaluate the fit of our focal model relative to the model with latitude and longitude coordinate pairs (average estimated concurvities of 0.19 and 0.54, respectively), we used blocked leave-one-out validation [76] at the level of counties. Using this method, RMSE for all estimates for our focal model and the model with latitude and longitude coordinate pairs were 0.07 and 0.08, respectively. This suggests that not including the thin plate spline across coordinate pairs results in little loss in terms of predictive power while also minimizing the possibility of over-fitting by reducing concurvity.

### Species-specific contributions to R_0_

Across the most sampled bird communities, no single bird species’ removal accounted for a median fold decrease in R_0_ larger than 0.92 or increase larger than 1.04. Mourning doves (*Zenaida macroura*, recorded in all bird communities) accounted for the largest dilution effect (median: 1.04-fold increase in R_0_, 1.01–1.11 in 95% of communities), while northern cardinals (*Cardinalis cardinalis*, recorded in 98.7% of the bird communities) accounted for the largest amplification effect (median: 0.92-fold decrease in R_0_, 0.83–0.99 in 95% of communities).

**Fig. 3:**
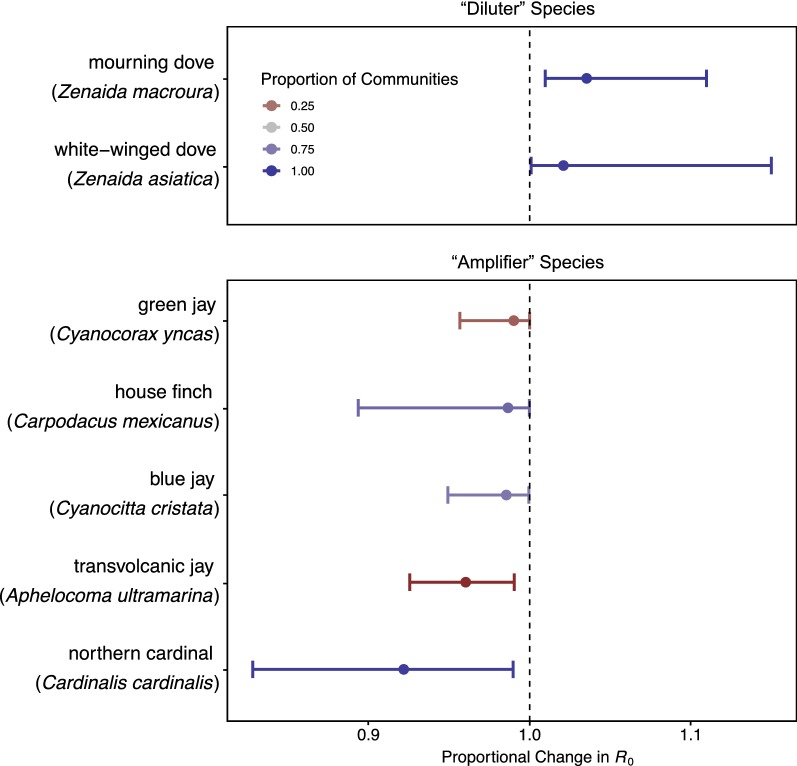
Keystone species. Bird species whose median estimates for their impact on R_0_ when they are removed from each community they occupy are greater than a 1.01 (dilution effect, the two species above the plot break in this figure), or less than a 0.99 (amplification effect, the four species below the plot break in this figure) fold change in R_0_. Intervals show median effects in 95% of the communities that each bird occupies

Only two species were estimated to have a median effect greater than a 1.01-fold increase in R_0_ [in order of median effect: mourning dove; white-winged dove (*Zenaida asiatica*)], and only five species had a median effect greater than a 0.99-fold decrease in R_0_ [in order of median effect: northern cardinal; transvolcanic jay (*Aphelocoma ultramarina*); blue jay (*Cyanocitta cristata*); house finch (*Carpodacus mexicanus*); green jay (*Cyanocorax yncas*)] (Fig. 3). Of the 15 most widespread species (species that appear in at least 95% of communities), the median estimate for five species was of an amplification effect. Eight of the fifteen species act as either “diluters” or “amplifiers” in at least 95% of communities, albeit with varying magnitudes. Of the 15 most abundant species (most individuals recorded; recorded in 34–99% of communities), the median effect on R_0_ for five species was below a ratio of one. Nine of these 15 species had an effect in 95% of communities on one side of a ratio of one.

Using a linear model with log of median bird relative abundance as a predictor for species physiological competence, physiological competence was predicted to increase with increasing relative abundance (estimate = 0.05, SE = 0.02, *t* = 3.00, *P <* 0.05). The estimate here refers to the increase in the number of infected mosquitoes with each unit increase of a bird’s relative abundance on the log scale (assuming a single mosquito bite per day over the course of a bird’s infectious period, which is generally assumed in measures of host competence [63]). We also found evidence for a negative relationship between bird species richness and community R_0_ using a linear model with log of species richness and temperature as predictors for median R_0_ and variation in R_0_ as weights (estimate: − 0.15, SE = 0.01, *t* = − 10.01, *P <* 0.05).

### Propagation of uncertainty

With no uncertainty propagated, median WNV R_0_ estimates were on average 1.03 times higher throughout the year and 1.06 times higher in the four most favorable months for transmission than in a model with all uncertainty propagated. Ignoring uncertainty had a much larger effect on variation among communities: CV in WNV R_0_ estimates were on average 1.32 times higher throughout the year and 1.56 times higher in the four most favorable months for transmission. This increase in magnitude and variation of the R_0_ estimates when no uncertainty was propagated is caused by the nonlinear averaging of variation in mosquito-to-bird transmission, mosquito survival, bird-to-mosquito transmission and bird survival. For example, translating the full distribution for bird’s titer profile (uncertainty) instead of a point estimate (no uncertainty), non-linearly, into the probability that a bird transmits infection to a susceptible mosquito given a bite homogenizes birds’ responses, decreasing variation among bird communities. This is a manifestation of Jensen’s inequality [98].

Species-specific contributions to R_0_ also depend on whether uncertainty is propagated. While the most influential bird species (northern cardinals and mourning doves) were robust to choices about uncertainty propagation, the ranks and identities of some of the top ten most important amplifier and diluter species changed.

### Complete eBird dataset

We present results using the complete eBird data set in Additional file 2: Figures S4, S5; Text S5, but suggest caution when drawing conclusions from these results because many of the estimates were obtained from poorly sampled bird communities. Using the complete eBird data resulted in greater variation in estimates for all outcomes: variation in R_0_ among communities increased (Additional file 2: Figures S4, S5), variation explained in the spatio-temporal GAM model decreased, and the estimated impacts of individual bird species on R_0_ were more extreme.

## Discussion

### Data limitations

Despite our ability to estimate R_0_ in individual bird communities, better data, such as mosquito populations on the same scale as the bird communities, are needed to make reliable quantitative estimates of WNV R_0_ across space and time. Given the size of our estimated effect of temperature on WNV transmission and the fact that different mosquito species incubate WNV and feed at different rates across temperatures [6, 14], variation in mosquito density and species composition among ecoregions and across seasons are likely the most important missing data needed to predict WNV R_0_ reliably. Our WNV R_0_ predictions for Texas counties relied on estimates of the mosquito-to-bird ratio and mosquito biting rate based on sparse data from a different geographical region (New Haven, CT, USA) and are assumed to be spatially and temporally homogeneous.

Though these simplifications result in an incomplete mechanistic model for WNV transmission, our model improves on previous models through its extensive use of empirical data, phylogenetic imputation to incorporate all birds within a community and treatment of both temperature-dependent mosquito incubation and survival. Many studies consider spatially (and/or temporally) variable mosquito densities, often in differential equation frameworks [12, 24, 25, 99–101]; however, these and other studies commonly ignore the effects of temperature on mosquito transmission probability. Other models consider variation in mosquito populations and temperature-dependent mosquito transmission probability, but use only a single class for “birds” and, like our study, a single type of mosquito (e.g. generically *Culex* sp.) [102, 103] (but see [104, 105] for two notable exceptions).

Importantly, all of these models use a tiny fraction of the available data; parameters are often informed by a single study and occasionally neglect uncertainty (this is most common in differential equation models, e.g. [24, 25, 99–101]). While little data and a simplified life-cycle (e.g. one bird species and temperature-independent mosquito-to-bird transmission) may allow models to examine, for example, the effects of different intervention strategies on R_0_ (e.g. [25, 100]), or the impact of seasonality in mosquito density (e.g. [102]) together these choices can increase (potentially dramatically) error in estimates for R_0_. Though we do our best to reduce error and over-confident estimates by using as much empirical data as possible over all aspects of the life-cycle of WNV, because we assume a constant mosquito-to-bird ratio (which assumes that the relative ratio of mosquito abundance to bird abundance is constant), mosquito biting rate and mosquito species composition, our estimates for WNV R_0_ are likely biased upwards in spring and winter months and underestimate the true variability in WNV R_0_ across space and time.

The first limitation arises because we assume a constant mosquito-to-bird ratio across months (the value we used is based on data collected in June and July [45]); we likely overestimated R_0_ in months with low mosquito density and possibly underestimated R_0_ in months with large mosquito populations. This assumption will have the largest influence in spring months when we estimated R_0_ transmission to be high because of a favorable temperature. In reality, small mosquito populations in these months probably result in lower WNV transmission. In the coldest winter months (e.g. December through February), our assumption of a constant mosquito-to-bird ratio is unlikely to change our estimate of WNV epidemic potential (R_0_ greater or less than one) in most counties because we already estimate most counties to have a low R_0_ because of unfavorable temperatures. However, in the warmest communities in winter months we estimated R_0_ to be greater than one (Fig. 1), which is unrealistic. Even in the absence of any Texas-specific *Culex* mosquito population data, data on mosquito populations across seasons from anywhere in the mid-west USA could potentially be used to reduce the number of implausible estimates (though it may be difficult to find good data on mosquito-to-bird ratios). As a first step, in Additional file 2: Figure S7 we show R_0_ estimates across months assuming the mosquito-to-bird ratio follows either a sinusoidal function or Gaussian function, with maxima in July and August, respectively. These results show, as expected, that strong seasonal variation in mosquito-to-bird ratio (with no uncertainty) constrains R_0_ estimates to resemble the assumed seasonal pattern in the mosquito-to-bird ratio.

Secondly, because we assume a constant mosquito-to-bird ratio and mosquito biting rate, our model undoubtedly underestimates the true variation in WNV R_0_ among Texas communities. For example, in October for all years between 2000–2017 we estimated that 95% of communities have an R_0_ between 1.40 and 2.51, with a SD among communities of 0.37. If we assume that the mosquito-to-bird ratio varies randomly across all Texas communities with a SD of 0.5, the R_0_ range spans 1.03 to 3.02 and the SD among R_0_ estimates increases to 0.55. Both mosquito population size and species composition are likely to vary predictably rather than randomly, such as systematic changes along a north to south or coastal to inland gradient (or correlated temperature gradients). Correlated spatial variation would cause further bias in our model estimates of R_0_. For example, if the mosquito-to-bird ratio is higher in coastal regions than in inland regions (mosquito density may be higher in coastal areas but it is unclear if, or how much, higher this ratio is), even if our assumed ratio of three mosquitoes per bird is an accurate representation of the mosquito-to-bird ratio on average, our R_0_ estimates will be too low in coastal regions and too high in inland regions. If mosquito species also vary in a spatially predictable way, any correlations between mosquito species and temperature would spatially bias our R_0_ estimates because of variation in WNV incubation rate among mosquito species [6].

Because of these possibilities, our model should not be taken as a complete, management-ready tool; the R_0_ values presented here should be interpreted with caution. With our model as is, more accurate R_0_ estimates could be obtained with higher-resolution mosquito data, which may be available for some locations outside of Texas. For example, the NEON (National Ecological Observation Network [106]) database provides mosquito sampling data for many locations across the USA; however, data for Texas was only available for two locations, and estimating mosquito-to-bird ratio from mosquito trapping data would require further simplifying assumptions [107]. In Europe, mosquito data are abundant in at least Italy and Germany (West Nile Disease National Surveillance Plan [108]). With these data and some additional data on the responses of European birds [38], our model could be extended to predict WNV transmission in Europe where human and equine cases of WNV are increasing [5, 8–12]. Alternatively, in the absence of mosquito data for a particular region, information on the ecological drivers of mosquito populations [109, 110] might be combined with data on habitat composition to estimate spatio-temporal, multi-species mosquito distributions.

A more fruitful approach may be to combine our data-rich analyses of the bird component of the life-cycle of WNV with the treatment given to the mosquito branch of the life-cycle in recent models such as those of Tran et al. [104], who consider variable host-vector contact rates across land cover types and seasons, and/or Marini et al. [12], who estimated WNV infection in an avian population in northern Italy with a model that includes temporally and spatially variable mosquito populations and temperature, as well as the effects of temperature on mosquito birth rate and bird-to-mosquito transmission.

Finally, limited spatial and temporal resolution in the eBird data was another constraint on our analysis. While eBird use is rapidly expanding, it may be worthwhile in the short term to incorporate bird abundance data from additional data sources such as the Breeding Bird Survey (BBS) or Christmas Bird Count (CBC), despite their more restricted seasonal coverage, or to use joint species distribution models to infer local bird community structure from habitat variables.

### Community R_0_

Though we neglect spatial variation in mosquito-to-bird ratio, mosquito biting rate and mosquito species, the single values that we used for these parameters result in estimates of WNV R_0_ that are similar to those of previous modeling efforts from other regions. For example, most of Hartley et al.’s [111] R_0_ estimates for California were between 1.0 and 1.75, while R_0_ estimates for New York City were 2.0 and 2.8 assuming mosquito-to-bird ratios of 2 and 4, respectively [112]. Finally, Wonham et al. [24] estimated that a mosquito-to-bird ratio of greater than 4.6 would have been required for the epidemic that occurred in New York, USA in 2000 (implying R_0_ = 1 for *M/B* = 4.6). Using our method, an R_0_ = 1 is obtained for *M/B* = 2.9 in the median county in July, though a ratio for *M/B* of only 2.0 is needed in the median county in May.

### Bird species-specific contributions to R_0_

At the level of individual bird species, some of our conclusions support the results of previous work, while others contradict previous findings. For example, Wonham et al. [24] assumed a *per capita* mosquito biting rate on American crows of 0.09 per day (CI: 0.03–0.16), which is similar to the biting rate we estimated for crows in our bird communities; our baseline biting rate of 0.14 per day and a median mosquito biting preference on American crows that is *≈* 1.8 times lower than on the average bird gives a biting rate of 0.08. Like previous syntheses (e.g. [1]), our model shows that species in the family Corvidae (e.g. jays, grackles and crows) are highly competent species for WNV. However, our model suggests that no single species ever accounts for more than approximately 30% of WNV R_0_, which contrasts with the results of [20] and [85] who found that more than 50% of infectious mosquitoes were infected by American robins, and [86] who found that 96% of mosquitoes were infected by either American robins or house sparrows (*Passer domesticus*). While these studies were conducted over a much smaller and almost entirely urban area with low bird diversity (90% of most of the bird communities sampled were composed of less than six species), the high proportion of mosquitoes infected by American robins which were present at a relative abundance between approximately 5–20% suggests that either: (i) we are missing an aspect of the interaction between WNV, mosquitoes and American robins; or (ii) our biological model is adequate and American robins are simply more important in other regions of the country.

To explore these two possibilities, we predicted the proportion of all newly infected mosquitoes attributable to each bird species in the community from [20] and [85] that had the highest proportion of American robins (7.5% of the bird community: Foggy Bottom neighborhood of Washington D.C, USA). For this community our median estimate for the proportion of all mosquitoes infected by American robins was 18%; however, uncertainty in mosquito biting preferences, bird species physiological competence and bird species detectability resulted in 95% confidence intervals spanning 3% to 79%. Three conclusions arise from the facts that the composition of mosquito blood meals observed in the Foggy Bottom neighborhood of Washington DC, USA by Kilpatrick et al. [20] is contained within our CI, and that American robins do not show up as one of the most important hosts in our communities. First, our model estimates are consistent with findings from a very different region of the country (albeit with very large uncertainty arising from propagating the uncertainty across the entire life-cycle of WNV). Secondly, regional differences in bird communities probably cause the differences in the estimated importance of American robins between our study and previous studies [85, 86]. Finally, regional and seasonal differences in mosquito feeding preferences [85, 113] probably also play an important role, reinforcing the need for more data on mosquitoes.

While estimated species-specific seroprevalence rates vary across studies, seroprevalence rates of northern cardinals are typically among the highest of all birds measured (Tammany Parish, LA [114]; Harris County, TX [115]; Illinois state-wide [116]; Chicago, IL [58]; Atlanta, GA [117]). High seroprevalence in northern cardinals suggests they may play a critical role in WNV amplification [114, 115], as we find here (Fig. 3). However, amplification within the bird community may or may not lead to higher human infection risk, and researchers disagree about the effect of northern cardinals on human infection risk [115, 117].

Previous studies have also found high seroprevalence for one of our most effective “diluter” species, the doves (family Columbidae). Rock pigeons (*Columba livia*) had one of the highest antibody prevalence rates in Georgia, USA between 2000 and 2004 [118], while mourning doves had the highest antibody prevalence rate in Chicago in 2005 and 2006 [58]. Our model shows that these species, which are strongly associated with urban landscapes and which we estimate to be among the least competent species for WNV, could be an important sink protecting human populations from disease. While we found only a small effect of decreasing R_0_ with increasing human population density (Fig. 2b), these species could potentially drive the result of Nolan et al. [16] that WNV *per capita* risk to humans decreased with increasing human population density.

### The dilution effect hypothesis

Studies testing the dilution effect hypothesis for WNV have obtained the full range of possible results: human cases declined with increasing bird diversity across 742 counties in 38 US states [28]; the proportion of mosquitoes infected with WNV declined with increasing diversity of non-passerine birds in Louisiana, USA [33]; Loss et al. [58] failed to detect a clear effect of species richness on WNV transmission in Chicago, IL, USA; Levine et al. [59] detected an amplification effect (overall seroprevalence increased with species diversity in Atlanta, GA, USA); in southern France [119] suggest that high bird diversity is a likely explanation of low numbers of horse infections, while [120] suggest that low number of human cases is due to the abundance of horses.

Based on the estimated competence of all 645 species found in the reduced eBird dataset and their median abundance in 2569 bird communities, host competence (the total number of mosquitoes that would be infected by an infected bird if it was bitten once each day of its infectious period [20, 63]) is positively correlated with relative abundance. Additionally, communities with higher species richness had a lower estimated R_0_, which is as expected if the most abundant birds are the most competent. These results support both a necessary condition (correlation between abundance and competence) and a primary expectation (correlation between richness and R_0_) of the dilution effect. However, we do not know what bird traits (or unobserved underlying ecological covariates) drive these patterns. To put it another way, we expect that R_0_ is proximally determined by the composition of the community, which is a function of many environmental covariates, rather than by species richness per se [56].

### Understanding spillover

Though we do not model human infections directly, we do find variation in WNV R_0_ among Texas bird communities that could shed light on patterns of human infection. According to [16] and [19], *per capita* infection risk is highest in northern Texas counties, with maximum risk in Castro, King, and Crosby counties. Two of these counties reside either entirely (Castro) or partially (Crosby) within the “High Plains” ecoregion of Texas, which we estimated to have the smallest R_0_ of all 11 ecoregions on average throughout the year. Unfortunately, we cannot validate these estimates in the absence of widespread spatial sampling of infected mosquitoes or birds. However, this apparent failure of our predictions (we expect human infection risk to be positively correlated with the R_0_ of WNV in local bird communities, but have no *a priori* expectation for the strength of this correlation) might be explained by variations in the degree of WNV spillover from birds to humans.

Spillover into human populations varies across microhabitats, seasons and mosquito communities [16, 28, 33, 84, 85, 121]. In Atlanta, GA, USA, for example, human infection rates are low despite similar mosquito infection rates and bird seroprevalence to other cities [117]. Levine et al. [117] attributed fewer human infections in Atlanta to high rates of infection in northern cardinals and blue jays, which they describe as “supersuppressor” species because they attract mosquito bites but fail to amplify transmission due to low competence. Our results (Fig. 3) and Komar et al. [114] suggested in contrast that northern cardinals and blue jays are important amplifier species (taking into consideration all experimental infections northern cardinals and blue jays are better defined as having moderate competence; their presence increases R_0_ within the bird community). Yet, it is still possible that the presence of these species could decrease the number of human cases by drawing mosquito bites, and hence infections, away from humans. Kilpatrick et al. [85] documented a related phenomenon, providing correlational evidence to suggest that higher numbers of human cases of WNV could be attributable to an increased number of human bites by *Culex* mosquitoes following seasonal emigration of American robins. Similarly, mosquito feeding on mammals increased in northern California following the fledging of ardeids (heron species) [113].

Our results, combined with the variation in previous results [16, 28, 33, 84, 114, 117, 121], bring into sharp focus how little we really know about the details of human infection risk across space and time in this system. To predict human infection cases for WNV, and for zoonotic diseases with heterogeneous host populations more generally, we envision a fine-scale spatial model that would use a Who Acquires Infection From Whom (WAIFW) matrix approach [122] and explicitly include humans as an additional species in the overall community. This framework would calculate the force of infection between each species pair, and could be used to determine the expected number of human cases during an epidemic. Interspecific contact rates could be parameterized using mosquito biting preferences, natural habitat type and land use (urban *vs* rural) on a very fine spatial scale. While eBird data is currently lacking to estimate the interface of bird communities with humans at a fine spatial scale for most locations, some counties in Texas (and other states) have thousands of complete lists submitted in spring and late summer months that could serve as model locations for analysis.

It is important to note, however, that recent progress has been made predicting human infection risk using alternative approaches to the data-driven heterogeneous transmission WAIFW approach we advocate. Using an ensemble forecasting framework informed by surveillance data of infected mosquitoes and humans, DeFelice et al. [123] produced good estimates for human cases of WNV in Long Island, New York despite using a very simple epidemiological model and assuming a constant mosquito population over outbreaks lasting approximately 20 weeks. Moon et al. [124] used an individual-based framework to predict human cases from California to New York in 2015 reasonably accurately despite relying solely on American robins as a measure of competent bird density. These results, while few, suggest that pursuing many different approaches may be the best method to improve our ability to estimate human infection.

### Propagation of uncertainty

Appropriate uncertainty and point estimates for R_0_ are only obtained when uncertainty in every sub-model is considered in calculations of R_0_. With the currently available data, we found large uncertainty in most of the models we use in our analysis, which obscures our ability to estimate R_0_ with precision in any individual community. While it is poor practice in general to use median estimates from models instead of all uncertainty, we examined the qualitative and quantitative effects of ignoring uncertainty in order to emphasize the importance of propagating uncertainty (and of reporting the procedures used). Ignoring uncertainty in our analyses would have led us to different quantitative and qualitative conclusions. Ignoring variation in sub-models increased variation in R_0_ estimates among communities for two related reasons. First, large uncertainty in birds’ physiological competence and mosquito biting preferences makes it more difficult to differentiate among birds, obscuring differences among communities. Secondly, birds are further homogenized due to the effects of Jensen’s inequality, which occurs when we transform the distribution of titer estimates into the probability that a bird transmits infection to a susceptible mosquito given a bite, which is bounded between zero and one. Jensen’s inequality also affects the estimated effects of temperature because of the nonlinear relationship between temperature and mosquito-to-bird transmission and mosquito survival, but has a larger effect when averaging temperature across either space or time (see Fig. 1).

In the absence of uncertainty, most bird species have an average titer that results in a bird-to-mosquito transmission probability beneath the inflection point of the logistic relationship between titer and transmission probability. Uncertainty in bird titer results in a non-negligible proportion of the posterior distribution for bird titer that is near or above 10^8^, which corresponds to a bird-to-mosquito transmission probability near one. This decreases variation in physiological competence among birds, which further narrows the variation in estimates among communities. This aspect of Jensen’s inequality will increase R_0_ estimates because of an increase in bird-to-mosquito transmission; however, increased titer will lead to lower bird survival, counteracting most, but not all, of this increase in R_0_ (60% of median estimates for each community were larger when uncertainty was not propagated). Because we were unable to estimate variation among species in mortality probability as a function of titer (that is, species variation in sensitivity to titer), estimated variation among birds is likely to be lower than true variation, further homogenizing birds and estimates among communities.

### WNV transmission in Europe

With additional data on the responses of European birds to WNV (e.g. [125–127]) and mosquito biting preferences and code modification, our model could be used to predict WNV transmission in many countries in Europe, with best results in those countries with abundant mosquito surveillance data (e.g. Germany, Italy [108]). Modeling studies on WNV spread in Europe have considered heterogeneities in mosquito transmission due to species [105] and temperature [29], as well as the effects of land cover and type on WNV transmission [119] and human infection risk [12]. However, like their North American counterparts, none of these studies consider full bird communities; our model can provide a method for incorporating heterogeneities in the bird community into spatio-temporal estimates of WNV transmission potential in Europe.

## Conclusions

Despite numerous data limitations at the scale we chose for our analyses, WNV remains a promising system for continued study on the mechanisms of vector-borne disease spillover on finer spatial scales. Using handpicked locations with sufficient bird community data, mosquito sampling and temperature variation, our modeling framework can be used as is to predict WNV R_0_ incorporating all known heterogeneities in transmission. With slight modifications, our model could be used to mechanistically estimate human infection probability as a function of bird community composition and other ecological predictors. We emphasize that a critical aspect of multi-faceted ecological analyses, such as modeling human infection risk to WNV, is transparency in model assumptions, choices and shortcomings; we hope that others will use our structure as a template for future analyses in order to increase model transparency.

## Additional files

**Additional file 1.** Code documentation. Code is available at: https://github.com/morgankain/WNV_Mechanistic_Model.

**Additional file 2.** Supplemental methods and results. **Text S1.** Methods: community resampling. **Figure S1.** RMSE and proportion of species absent from 100 subsamples from each of the 46 eBird communities with greater than 1300 complete lists. **Figure S2.** Gain in RMSE and loss of species with increasing retention of Texas bird communities as a consequence of less stringent criteria defining what is a “well sampled” bird community. **Figure S3.** Delineation of the area where eBird lists were used as prior information for the bird community sampled in [45]. **Text S2.** Methods: phylogenetic mixed effects model validation. **Table S1.** Phylogenetic mixed effects model validation. We used RMSE to quantify leave-one-out cross validation error for titer, biting preference, and detectability models, and AUC to quantify error for the survival model. **Text S3.** Ricker function. **Text S4.** Results: Community R_0._ **Figure S4.** A comparison of WNV R_0_ estimates between models fit to the full and reduced eBird datasets. **Figure S5.** Histogram of R_0_ estimates among Texas counties for the full and reduced eBird datasets. **Figure S6.** WNV R_0_ estimates for Texas counties when no uncertainty is propagated. **Figure S7.** WNV R_0_ estimates for Texas counties assuming a seasonally varying mosquito-to-bird ratio. **Text S5.** Results: species specific-contributions to R_0._ Results for the complete Texas eBird dataset. **Text S6.** Data citations. Publications from which data was obtained.

## Data Availability

All data and extensively commented R code used in this study are available at: https://github.com/morgankain/WNV_Mechanistic_Model.

## Abbreviations

CV: coefficient of variation
GAM: generalized additive model
GLMM: generalized linear mixed effects model
IUCN: International Union for Conservation of Nature
MAD: mean absolute deviation
NOAA: National Oceanic and Atmospheric Administration
RMSE: root mean squared error
SD: standard deviation
vcov: variance-covariance
WAIFW: Who Acquires Infection From Whom
WNV: West Nile virus
