# Supplementary material for: Predicting West Nile virus transmission in North American bird communities using phylogenetic mixed effects models and eBird citizen science data
Source: Parasit Vectors. 2019 Aug 8;12:395. doi: 10.1186/s13071-019-3656-8 (PMC6686473; doi:10.1186/s13071-019-3656-8)
Supplement: Supplementary file 1 — Additional file 1. Code documentation. Code is available at: https://github.com/morgankain/WNV_Mechanistic_Model. [file 13071_2019_3656_MOESM1_ESM.docx]

All R code and data required to run the analyses presented in this Parasites & Vectors paper (Kain and Bolker: Predicting West Nile virus transmission in North American bird communities using phylogenetic mixed effects models and eBird citizen science data) are available at: https://github.com/morgankain/WNV_Mechanistic_Model

------------------------------------

Full details are provided in that repo. However, in brief:

The code can be run using two methods:

1) Clean eBird .zip file and run R code "manually" in two separate steps

A) Run sh ebird_data_clean.sh in the command prompt once all eBird .zip files have been placed in the folder titled "ebird_zip_fresh". Note: Requires "Command Line Tools" on Mac.

B) Open "top_level_script.R". Details for the purpose of each R script can be found in "top_level_script.R"

i) Read the instructions at the top of this script

ii) Adjust parameters and options as desired

iii) Run the script as desired

2) Clean eBird .zip file and run R code in a single step

A) Run sh ebird_bash_run.sh

i) This method will require some additional setup on the part of the user (see Step 4 in the file: "ebird_bash_run.sh")

ii) This method can't be used if species scientific names are mismatched (see "saved_matching" below)

This pipeline contains and requires a number of folders, many of which begin empty. The folders are one of three types:

1) Folders that need to have data placed in them prior to running the code.

A) trees -- Contains phylogenetic tree data

B) ebird_zip_fresh -- Contains the .zip ebird file

C) data -- Contains all other data (bird responses, county data etc.)

2) Folders that contain model components

A) stan -- Contains stan model definitions

3) Folders that start empty but get filled as part of the automated workflow when output is saved to disk

A) ebird_data_for_R -- Will contain all of the eBird data once it is extracted

B) ebird_zip_dump -- Will contain .zip files after they get extracted

C) ebird_pieces -- One of two intermediate folders to momentarily house extracted eBird pieces

D) ebird_unzip -- Two of two intermediate folders to momentarily house extracted eBird pieces

E) saved_fits -- Houses intermediate fitted model results to expedite code in future runs

F) saved_matching -- Houses matched scientific names (between eBird and the bird phylogeny) and bird body sizes

G) saved_output -- Houses all other fits including final "product"

Again, Details for the purpose of each R script can be found in "top_level_script.R"
