# Supplementary material for: Predicting West Nile virus transmission in North American bird communities using phylogenetic mixed effects models and eBird citizen science data
Source: Parasit Vectors. 2019 Aug 8;12:395. doi: 10.1186/s13071-019-3656-8 (PMC6686473; doi:10.1186/s13071-019-3656-8)
Supplement: Supplementary file 2 — Additional file 2. Supplemental methods and results. Text S1. Methods: community resampling. Figure S1. RMSE and proportion of species absent from 100 subsamples from each of the 46 eBird communities with greater than 1300 complete lists. Figure S2. Gain in RMSE and loss of species with increasing retention of Texas bird communities as a consequence of less stringent criteria defining what is a “well sampled” bird community. Figure S3. Delineation of the area where eBird lists were used as prior information for the bird community sampled in [45]. Text S2. Methods: phylogenetic mixed effects model validation. Table S1. Phylogenetic mixed effects model validation. We used RMSE to quantify leave-one-out cross validation error for titer, biting preference, and detectability models, and AUC to quantify error for the survival model. Text S3. Ricker function. Text S4. Results: Community R0. Figure S4. A comparison of WNV R0 estimates between models fit to the full and reduced eBird datasets. Figure S5. Histogram of R0 estimates among Texas counties for the full and reduced eBird datasets. Figure S6. WNV R0 estimates for Texas counties when no uncertainty is propagated. Figure S7. WNV R0 estimates for Texas counties assuming a seasonally varying mosquito-to-bird ratio. Text S5. Results: species specific-contributions to R0. Results for the complete Texas eBird dataset. Text S6. Data citations. Publications from which data was obtained. [file 13071_2019_3656_MOESM2_ESM.pdf]

Predicting West Nile virus transmission in North American bird communities using phylogenetic mixed effects models and eBird citizen science data

Morgan P. Kain[1] **and Benjamin M. Bolker**[1,2]

[1]**Department of Biology, McMaster University, 1280 Main Street West, Hamilton, Ontario L8S 4K1 Canada**
[2]**Department of Mathematics and Statistics, McMaster University, 1280 Main Street West, Hamilton, Ontario**

Author's Email Addresses:
Corresponding Author: Morgan P. Kain (kainm@mcmaster.ca)
Benjamin M. Bolker (bolker@mcmaster.ca)

Text S1. Methods: community resampling

Here we present the results of our simulated resampling of the best sampled bird communities in Texas, USA in the eBird database (communities with greater than 1,300 complete lists). We resampled 5-120 complete lists from the 46 most sampled communities 100 times. From these results we determined, subjectively, that with fewer than 80 complete lists, the gain in total number of communities was not worth the increased error rate and loss of species representation; at greater than 80 complete lists the loss in communities was too large for the small decrease in error and small gain in species.

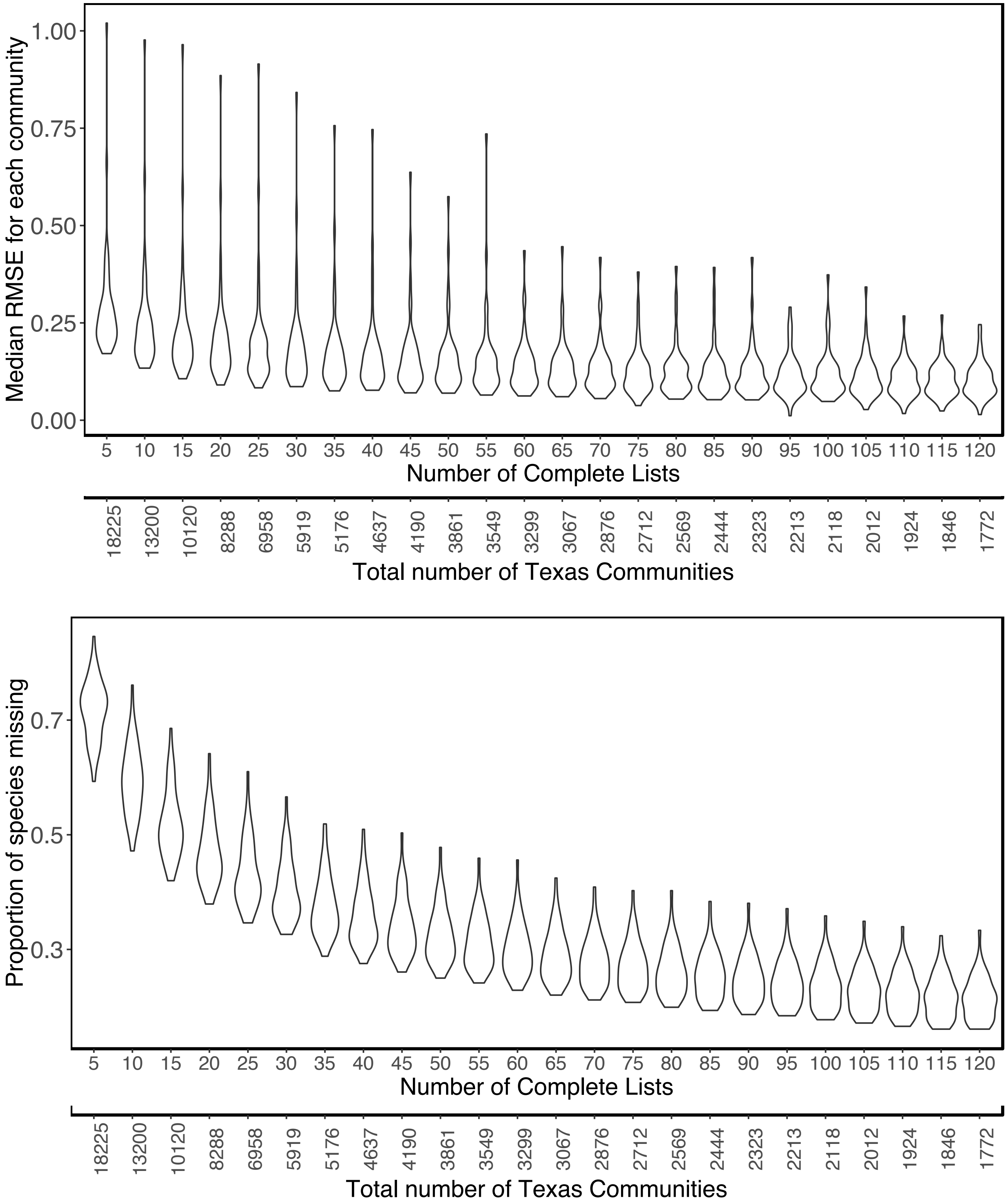

Figure S1. Result from 100 subsamples from each of the 46 communities with greater than 1,300 complete lists. Violin plots show distribution of medians across the 46 communities from the 100 resampling events. The top panel shows the RMSE between the relative proportions of all birds in the complete communities and subsampled communities as a function of the number of lists subsampled. The secondary axis shows the number of communities that would be left in the Texas dataset if the corresponding number of lists were used as a cutoff to define a well sampled community.

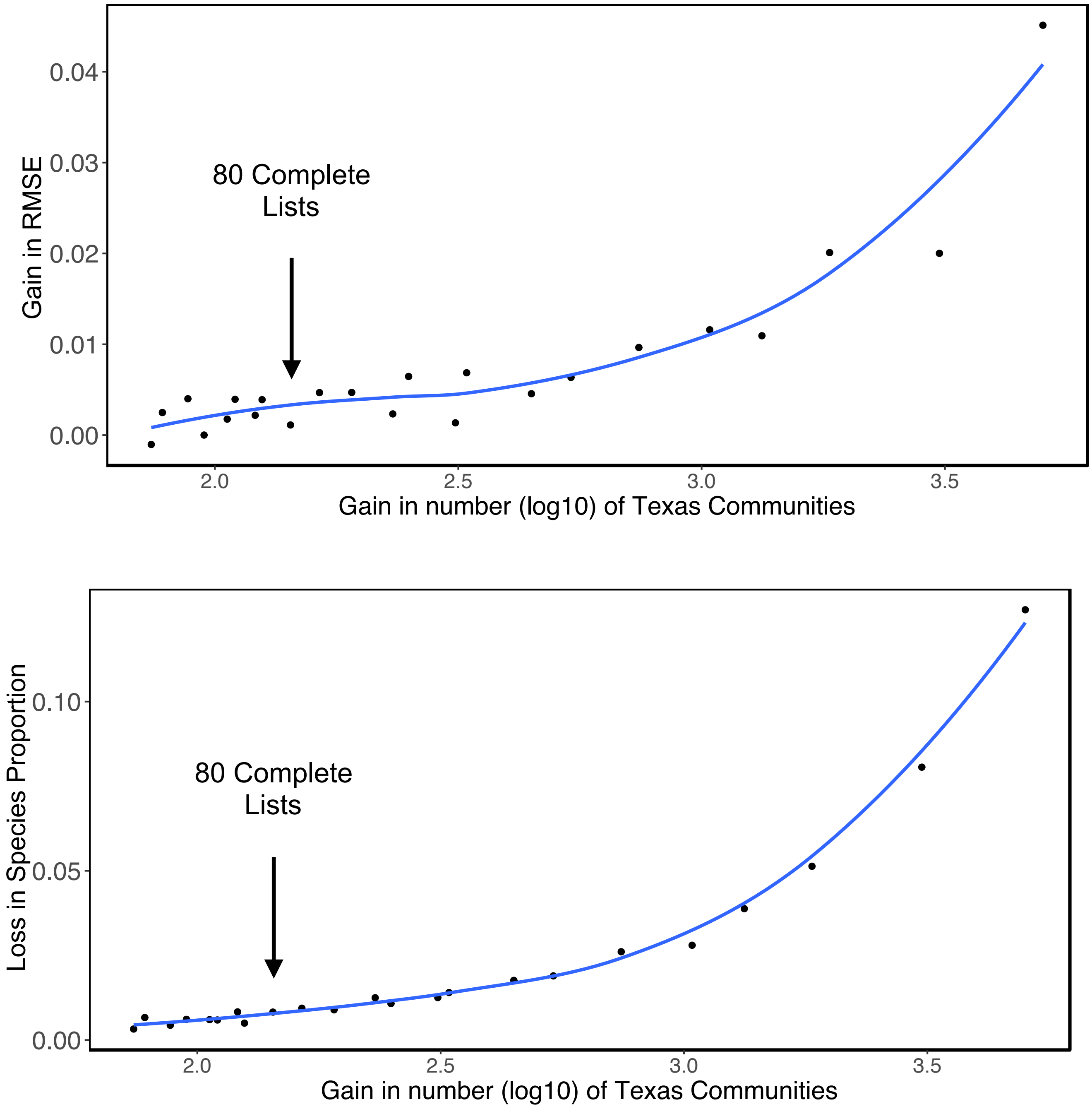

Figure S2. A second depiction of results from 100 subsamples from each of the 46 communities with greater than 1,300 complete lists. Top and bottom panels shows how much RMSE is gained, or the additional proportion of species that are lost, respectively, with an increase in retained Texas communities (which are gained when fewer complete lists are used as a cutoff to define a well sampled community, see Figure S1). Points in both panels show overall medians across all communities and 100 resampling events. Blue lines are smooths showing approximate slope. 80 complete lists resides at approximately the beginning of the accelerating portion of this curve, though our overall choice of 80 communities is fundamentally a qualitative and not quantitative decision.

Sampling region for the mosquito biting preferences model

Figure S3 shows the eBird sampling region used to inform our prior in the Bayesian component of the mosquito biting preference model.

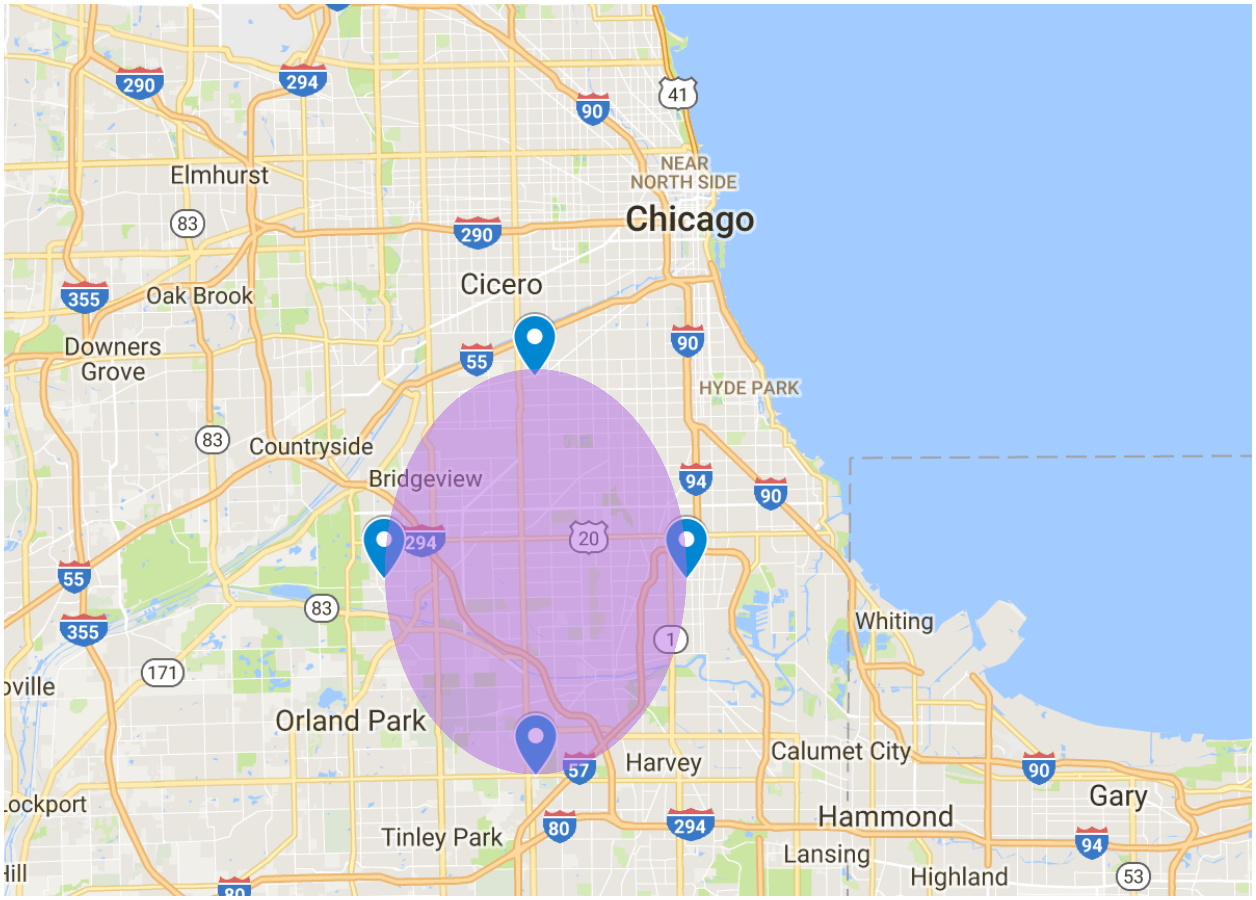

Figure S3. Delineation of the area where eBird lists were used as prior information for the bird community sampled in [1]. The center of this region was given as the location of the sampling conducted in [1]. The entire area is contained within Cook County, IL and is composed primarily of suburban Chicago, IL.

Text S2. Methods: phylogenetic mixed effects model validation

We present results for R^2 and blocked leave-one-out cross validation in Table S1. We calculated conditional R^2 (which measures the variance explained by both fixed and random factors; see [2, 3, 4]) for our phylogenetic mixed models using slightly modified code from `R` package `MuMIn` [5]). For blocked leave-one-out cross validation we left out all data for a single species at a time, refit our model with and without the species level phylogenetic random effect, and calculated goodness of fit of predicted values to the left out bird species' data for each model. For models with a normal or Poisson error distribution we use scaled root mean squared error (RMSE) between predicted values and the data with the following formula:

$$\frac{\sqrt{\frac{1}{n}\sum_{i=1}^{n}\left(y_i-\hat{y}_i\right)^2}}{\bar{y}}. \tag{1}$$

For predicting responses using the titer model we use mean log dose across all infection experiments (which decreases fit to individual infection experiments, inflating RMSE slightly). For the binomial model (bird survival), we use area under the curve (AUC), calculated using the observed number of birds that died and did not die on each day and the estimated probabilities for each of these outcomes. Set up in this way, larger AUC values mean a better fit between the predicted values and the empirical data. AUC cannot be calculated for scenarios in which no birds died; in our calculation we ignore experiments in which no individual birds died. To calculate AUC we used the `R` package `MESS` [6]. Table S1 shows mean RMSE across all species for each method.

Despite an established method for calculating R^2 for mixed effects models [2, 3, 4], R^2 can be difficult to interpret for these models. Additionally, by convention, the biting preference model without the phylogenetic random effect (an intercept only model) has an $R^2 = 0$, which makes the ratio of R^2 values for this model difficult to interpret. The important point is that R^2 increases (in many cases greatly) for all four of our models when the species level phylogenetic random effect is included (Table S1).

The inclusion of a species level phylogenetic random effect increases the ability of three of four models to predict left-out species' responses (Table S1). For the survival model, AUC values are small overall because most bird species survive with high probability; it is difficult to predict the rare case of individual birds dying. AUC increases when a phylogenetic random effect is included in the survival model because this model is better able to predict the mortality of species that die with a higher frequency than the average species. For the biting preference model, the phylogenetic random effect marginally increases RMSE (Table S1); this may be due to minor overfitting.

Table S1. Phylogenetic mixed effects model validation. We use RMSE to quantify leave-one-out cross validation error for titer, biting preference, and detectability models, and AUC to quantify error for the survival model.

	**Model**							
	**Titer**		**Survival**		**Biting preference**		**Detectability**	
**Phylogenetic random effect included?**	No	Yes	No	Yes	No	Yes	No	Yes
Conditional R^2	0.65	0.93	0.805	0.898	0.00	0.97	0.51	0.92
Leave-one-out goodness of fit estimate	0.48	0.41	0.01	0.03	0.23	0.30	0.25	0.18

Text S3. Methods: Ricker function

The Ricker function:

$$y = axe^{-bx} \tag{2}$$

is a hump-shaped curve that is commonly used for modeling right-skewed patterns in ecology [7] such as density dependence (e.g. [8]). When $a = 1$ this function is a Ricker function; when $a \neq 0$ this function is a Generalized Ricker. We use a Generalized Ricker function to model patterns in titer in birds, where x is given by day. Using log(titer) as our response variable, we estimate the a and b parameters in the Generalized Ricker function using a linear model with the form log(titer) $\sim$ day + log(day).

Text S4. Results: community $\mathcal{R}_0$

Complete eBird Dataset

Estimates for WNV $\mathcal{R}_0$ using the complete eBird dataset were more variable, but had similar monthly medians to the well sampled eBird dataset in most months (Figure S4, Figure S5).

The spatio-temporal GAM model explained 97% of the variation in WNV $\mathcal{R}_0$. WNV $\mathcal{R}_0$ was once again found to be driven mostly by temperature, and vary little across human population density and years. With the full dataset, bird communities most favorable for WNV transmission were once again estimated to be in the "Llano Uplift" ecoregion, while the least competent bird communities were estimated to be in the "High Plains" and "Oak Woods & Prairies" ecoregions. Overall $\mathcal{R}_0$ was estimated to be the highest in the "Piney Woods" and "Oak Woods & Prairies" (favorable temperature in the "Oak Woods & Prairies" has a much larger effect than the unfavorable bird communities in this region), and the lowest in the "High Plains".

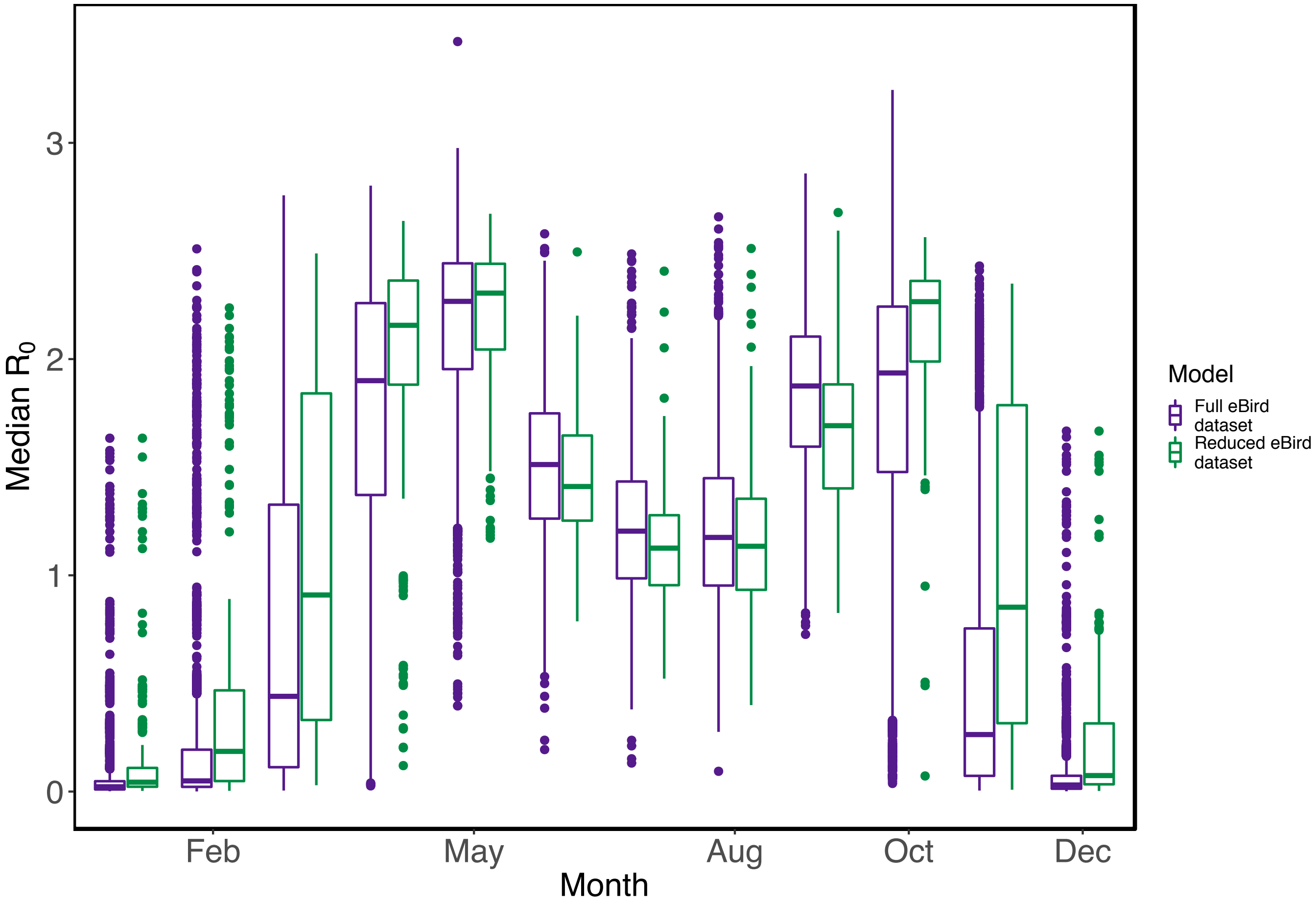

Figure S4. A comparison of $\mathcal{R}_0$ estimates. Purple and green boxplots show $\mathcal{R}_0$ estimates from models fit to the full and reduced eBird datasets respectively.

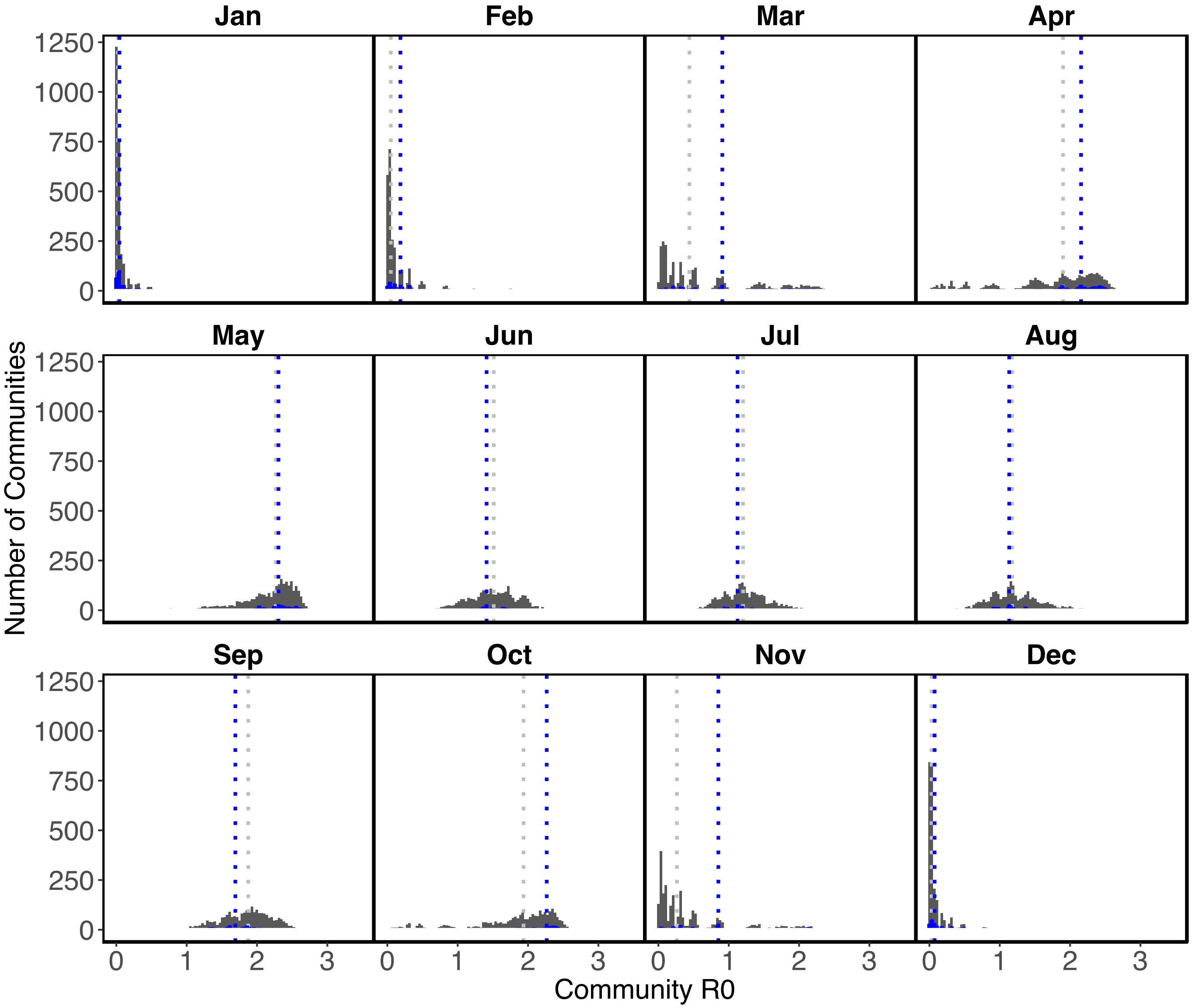

Figure S5. A comparison of $\mathcal{R}_0$ estimates. The gray histograms shows estimates for the full ebird data set and the blue histograms shows estimates for the reduced eBird data set by month. The vertical dotted gray and blue lines show the medians of the distributions.

No uncertainty propagated: reduced eBird Dataset

Estimates for WNV $\mathcal{R}_0$ when propagating no uncertainty were more variable than when all uncertainty was propagated (Figure S6 vs main text Figure 1). No uncertainty propagation increased the impact of variation in the bird community on variation in $\mathcal{R}_0$ estimates within months (Figure S6).

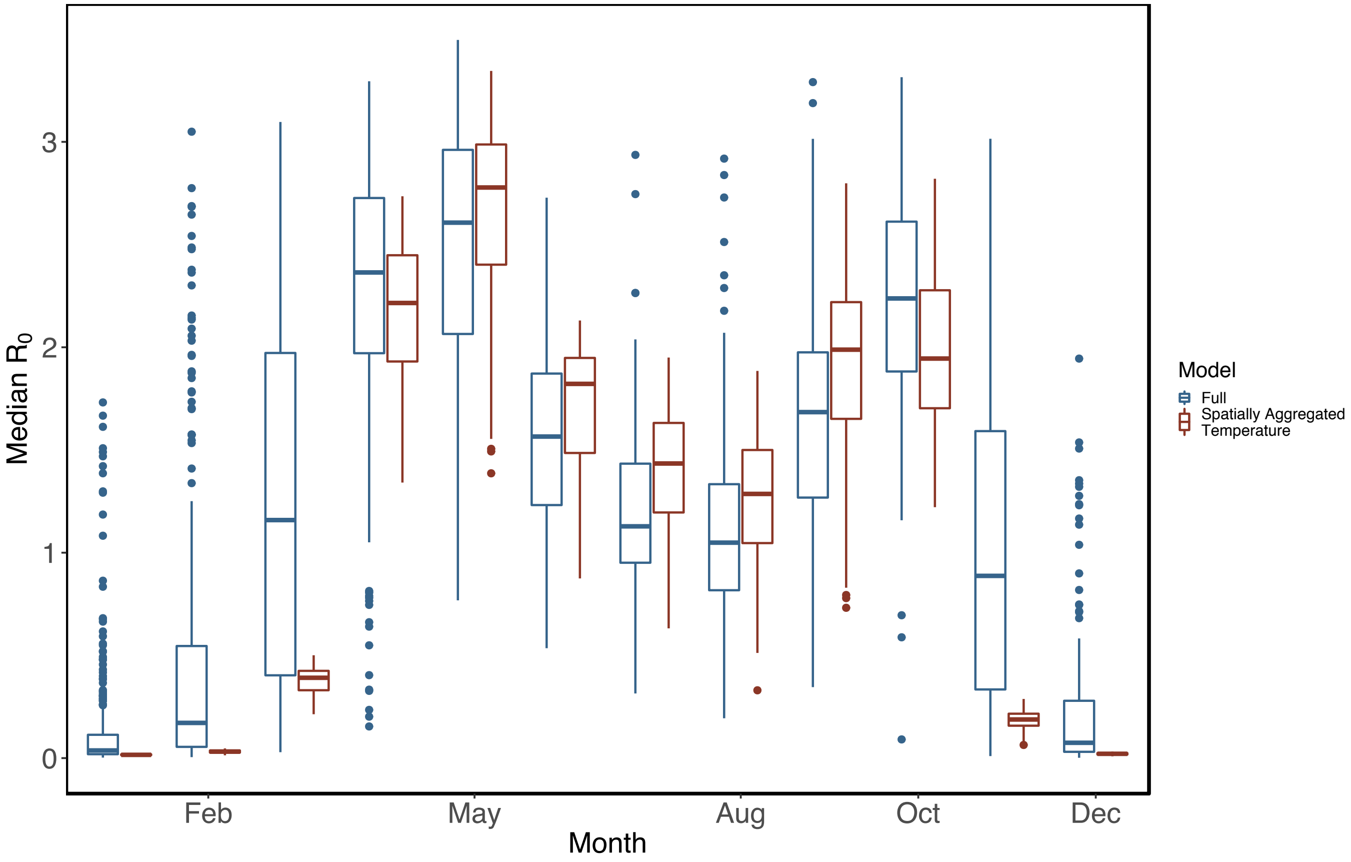

Figure S6. WNV $\mathcal{R}_0$ estimates between months and among Texas counties when no uncertainty is propagated. Blue boxplots show $\mathcal{R}_0$ estimates across Texas counties within months for a "Full" model, which used the eBird community and NOAA temperature data for each community. Red boxplots show $\mathcal{R}_0$ estimates from a model where each community retained their specific eBird community, but whose temperature was replaced with average temperature over all of Texas for that month. With no uncertainty propagated, variation in $\mathcal{R}_0$ within months attributable to variation in the bird communities (red boxplots) is much larger than when all uncertainty is propagated (see main text Figure 1). The increase in variation explained by the bird community is due to the translation of titer into bird-to-mosquito transmission probability, which has a maximum of one (see main text: Discussion for a full description of this phenomenon). Increases or decreases in medians between the models within months is due to the effects of averaging temperature prior to predicting $\mathcal{R}_0$ using the nonlinear functions for mosquito-to-bird transmission and mosquito survival across temperature.

Seasonally variable mosquito-to-bird ratio

Estimates for WNV $\mathcal{R}_0$ with seasonally vairable mosquito-to-bird ratio are shown in Figure S7. With strong seasonal variation in mosquito-to-bird ratio that follows either a sinusoidal function or a Gaussian function with peaks in July or August respectively, estimates for $\mathcal{R}_0$ are lower in the spring months, and lower overall than the estimates we obtain using our full model.

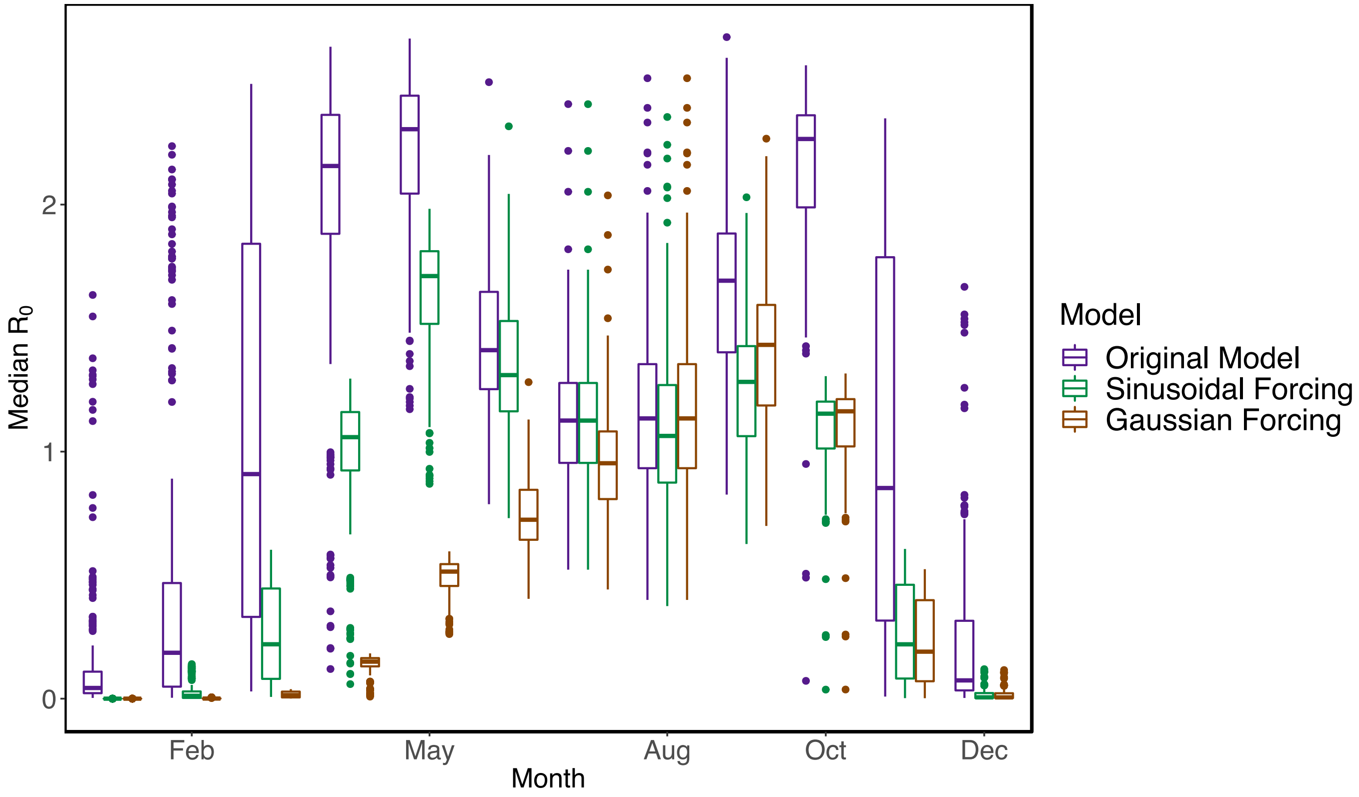

Figure S7. WNV $\mathcal{R}_0$ estimates between months and among Texas counties. Purple boxplots show $\mathcal{R}_0$ estimates across Texas counties within months for our full model, which used the eBird community and NOAA temperature data for each community, but assumes a constant mosquito-to-bird ratio across space and time. Green boxplots use the same model but assume that mosquito-to-bird ratio follows a cyclic sinusoidal function with a peak in mid July and a trough in mid February. Orange boxplots use the same model but assume that mosquito-to-bird ratio follows a Gaussian function with a peak in August. Given these hypothetical seasonal patterns in mosquito-to-bird ratio, assuming a constant mosquito-to-bird ratio (purple boxplots) results in higher $\mathcal{R}_0$ estimates in every month.

Single sources of uncertainty: reduced eBird Dataset

For each model and for most sources of uncertainty, using point estimates for a single parameter decreases the CV of $\mathcal{R}_0$ and increases the amount of variance explained in the spatio-temporal model. However, for the titer model, ignoring uncertainty in fixed effects increases the CV because of a smaller decrease in standard deviation than in the mean. In the titer model, ignoring uncertainty in the amount of evolutionary change along the most recent branch in time leading to species i results in the largest change in the CV of $\mathcal{R}_0$ for any source of uncertainty for any model, regardless of direction: a median of a 1.3 fold increase in the CV of $\mathcal{R}_0$.

Text S5. Results: species-specific contributions to $\mathcal{R}_0$

Complete eBird Dataset

Here we present which species have the largest impact on $\mathcal{R}_0$ when they are removed from each of the 30,188 communities that they occupy, which gives a distribution of effects across communities. Northern cardinals were estimated to have a median effect on $\mathcal{R}_0$ similar in magnitude to their effect in the reduced dataset, but with a larger range of effects (recorded in 68% of the bird communities in the full dataset; median: 0.91 fold decrease in $\mathcal{R}_0$, 0.76-0.99 in 95% of communities). Jay species appear as the most impactful amplifiers in the full dataset as well. As in the reduced data set, Mourning doves (*Zenaida macroura*, recorded in 78% of the bird communities in the full dataset) accounted for the largest median dilution effect, but with an increased range of effect sizes (median: 1.05 fold increase in $\mathcal{R}_0$, 1.01-1.30 in 95% of communities). Again, only 2 species (Mourning doves: *Zenaida macroura* and White-winged doves: *Zenaida asiatica*) had a median dilution effect greater than a 1.01 fold increase in $\mathcal{R}_0$.

These analyses also provide a clear example of the type of problems that arise when using undersampled bird communities. In November of 2004 in Lamb county, a single complete list was submitted with two species, Sharp-tailed sandpiper (*Calidris acuminata*) and Ring-necked pheasant (*Phasianus colchicus*). Sharp-tailed sandpipers are estimated to be moderately competent hosts, while Ring-necked pheasants are estimated to be very poor hosts. This is the only "community" in which Sharp-tailed sandpipers were recorded in all of Texas. Sharp-tailed sandpipers were estimated to have a dilution effect of 0.29, which is implausible given the magnitude of the effects we estimate for the most important amplifier and diluter species.

Text S6. data citations

Laboratory infections of birds: [9, 10, 11, 12, 13, 14, 15, 16, 17, 18, 19, 20, 21, 22, 23, 24, 25, 26, 27, 28, 29, 30, 31, 32, 33, 34, 35, 36, 37, 38]

Laboratory infections of mosquitoes: bird-to-mosquito and mosquito-to-bird transmission: [35, 37, 39, 40, 41, 42, 43, 44, 45, 46, 47, 48, 49, 50, 51, 52, 53, 54, 55, 56, 57, 58, 59, 60, 61, 62, 63, 64, 65, 66, 67, 68, 69]

Bird detectability: [70, 71, 72, 73], and [74, 75, 76, 77, 78, 79, 80] (cited in [81])

References

[1] Hamer GL, Kitron UD, Goldberg TL, Brawn JD, Loss SR, Ruiz MO, et al. Host selection by *Culex pipiens* mosquitoes and West Nile virus amplification. The American Journal of Tropical Medicine and Hygiene. 2009;80(2):268–278.

[2] Nakagawa S, Schielzeth H. A general and simple method for obtaining R2 from generalized linear mixed-effects models. Methods in Ecology and Evolution. 2013;4(2):133–142.

[3] Johnson PC. Extension of Nakagawa & Schielzeth's R2GLMM to random slopes models. Methods in Ecology and Evolution. 2014;5(9):944–946.

[4] Nakagawa S, Johnson PC, Schielzeth H. The coefficient of determination R2 and intra-class correlation coefficient from generalized linear mixed-effects models revisited and expanded. Journal of the Royal Society Interface. 2017;14(134):20170213.

[5] Barton K, Barton MK. Package 'MuMIn'. 2018;.

[6] Ekstrøm CT. MESS: Miscellaneous Esoteric Statistical Scripts; 2019. R package version 0.5.5. Available from: https://CRAN.R-project.org/package=MESS.

[7] Bolker BM. Ecological models and data in R. Princeton, NJ: Princeton University Press; 2008.

[8] Elliott J. Mechanisms responsible for population regulation in young migratory trout, *Salmo trutta*. The critical time for survival. The Journal of Animal Ecology. 1989;p. 987–1001.

[9] Brault AC, Langevin SA, Bowen RA, Panella NA, Biggerstaff BJ, Miller BR, et al. Differential virulence of West Nile strains for American crows. Emerging Infectious Diseases. 2004;10(12):2161.

[10] Brault AC, Huang CY, Langevin SA, Kinney RM, Bowen RA, Ramey WN, et al. A single positively selected West Nile viral mutation confers increased virogenesis in American crows. Nature Genetics. 2007;39(9):1162–1166.

[11] Brault AC, Langevin SA, Ramey WN, Fang Y, Beasley DW, Barker CM, et al. Reduced avian virulence and viremia of West Nile virus isolates from Mexico and Texas. The American Journal of Tropical Medicine and Hygiene. 2011;85(4):758–767.

[12] Clark L, Hall J, McLean R, Dunbar M, Klenk K, Bowen R, et al. Susceptibility of greater sage-grouse to experimental infection with West Nile virus. Journal of Wildlife Diseases. 2006;42(1):14–22.

[13] Duggal NK, Bosco-Lauth A, Bowen RA, Wheeler SS, Reisen WK, Felix TA, et al. Evidence for co-evolution of West Nile Virus and house sparrows in North America. PLoS Neglected Tropical Diseases. 2014;8(10):e3262.

[14] Fang Y, Reisen WK. Previous infection with West Nile or St. Louis encephalitis viruses provides cross protection during reinfection in house finches. The American Journal of Tropical Medicine and Hygiene. 2006;75(3):480–485.

[15] Grubaugh ND, Smith DR, Brackney DE, Bosco-Lauth AM, Fauver JR, Campbell CL, et al. Experimental evolution of an RNA virus in wild birds: evidence for host-dependent impacts on population structure and competitive fitness. PLoS Pathogens. 2015;11(5):e1004874.

[16] Guerrero-Sánchez S, Cuevas-Romero S, Nemeth NM, Trujillo-Olivera M, Worwa G, Dupuis A, et al. West Nile virus infection of birds, Mexico. Emerging Infectious Diseases. 2011;17(12):2245–2252.

[17] Hofmeister E, Porter RE, Franson JC. Experimental susceptibility of wood ducks (Aix sponsa) for West Nile virus. Journal of wildlife diseases. 2015;51(2):411–418.

[18] Hofmeister EK, Lund M, Shearn-Bochsler V, Balakrishnan CN. Susceptibility and antibody response of the laboratory model zebra finch (Taeniopygia guttata) to West Nile virus. PloS one. 2017;12(1):e0167876.

[19] Kilpatrick AM, Dupuis AP, Chang GJJ, Kramer LD. DNA vaccination of American robins (*Turdus migratorius*) against West Nile virus. Vector-Borne Zoonot. 2010;10(4):377–380.

[20] Kilpatrick AM, Peters RJ, Dupuis AP, Jones MJ, Daszak P, Marra PP, et al. Predicted and observed mortality from vector-borne disease in wildlife: West Nile virus and small songbirds. Biological Conservation. 2013;165:79–85.

[21] Kinney RM, Huang CYH, Whiteman MC, Bowen RA, Langevin SA, Miller BR, et al. Avian virulence and thermostable replication of the North American strain of West Nile virus. Journal of General Virology. 2006;87(12):3611–3622.

[22] Kipp AM, Lehman JA, Bowen RA, Fox PE, Stephens MR, Klenk K, et al. West Nile virus quantification in feces of experimentally infected American and fish crows. The American Journal of Tropical Medicine and Hygiene. 2006;75(4):688–690.

[23] Komar N, Langevin S, Hinten S, Nemeth N, Edwards E, Hettler D, et al. Experimental infection of North American birds with the New York 1999 strain of West Nile virus. Emerging Infectious Diseases. 2003;9(3):311–322.

[24] Komar N, Panella NA, Langevin SA, Brault AC, Amador M, Edwards E, et al. Avian hosts for West Nile virus in St. Tammany Parish, Louisiana, 2002. The American Journal of Tropical Medicine and Hygiene. 2005;73(6):1031–1037.

[25] Langevin SA, Brault AC, Panella NA, Bowen RA, Komar N. Variation in virulence of West Nile virus strains for house sparrows (*Passer domesticus*). The American Journal of Tropical Medicine and Hygiene. 2005;72(1):99–102.

[26] Langevin SA, Bowen RA, Reisen WK, Andrade CC, Ramey WN, Maharaj PD, et al. Host competence and helicase activity differences exhibited by West Nile viral variants expressing NS3-249 amino acid polymorphisms. PLoS ONE. 2014;9(6):e100802.

[27] Melian EB, Hall-Mendelin S, Du F, Owens N, Bosco-Lauth AM, Nagasaki T, et al. Programmed ribosomal frameshift alters expression of West Nile Virus genes and facilitates virus replication in birds and mosquitoes. PLoS Pathogens. 2014;10(11):e1004447.

[28] Nemeth NM, Hahn DC, Gould DH, Bowen RA. Experimental West Nile virus infection in eastern screech owls (*Megascops asio*). Avian Diseases. 2006;50(2):252–258.

[29] Nemeth NM, Oesterle PT, Bowen RA. Humoral immunity to West Nile virus is long-lasting and protective in the house sparrow (*Passer domesticus*). The American Journal of Tropical Medicine and Hygiene. 2009;80(5):864–869.

[30] Nemeth N, Thomsen B, Spraker T, Benson J, Bosco-Lauth A, Oesterle P, et al. Clinical and pathologic responses of American crows (*Corvus brachyrhynchos*) and fish crows (*C. ossifragus*) to experimental West Nile virus infection. Veterinary Pathology. 2011;48(6):1061–1074.

[31] Owen J, Moore F, Panella N, Edwards E, Bru R, Hughes M, et al. Migrating birds as dispersal vehicles for West Nile virus. EcoHealth. 2006;3(2):79–85.

[32] Owen JC, Nakamura A, Coon CA, Martin LB. The effect of exogenous corticosterone on West Nile virus infection in Northern Cardinals (*Cardinalis cardinalis*). Veterinary Research. 2012;43(1):34.

[33] Reisen WK, Fang Y. Does feeding on infected mosquitoes (Diptera: Culicidae) enhance the role of song sparrows in the transmission of arboviruses in California? Journal of Medical Entomology. 2007;44(2):316–319.

[34] Reisen WK, Hahn DC. Comparison of immune responses of brown-headed cowbird and related blackbirds to West Nile and other mosquito-borne encephalitis viruses. Journal of Wildlife Diseases. 2007;43(3):439–449.

[35] Reisen W, Fang Y, Martinez V. Avian host and mosquito (Diptera: Culicidae) vector competence determine the efficiency of West Nile and St. Louis encephalitis virus transmission. Journal of Medical Entomology. 2005;42(3):367–375.

[36] VanDalen KK, Hall JS, Clark L, McLean RG, Smeraski C. West Nile virus infection in American robins: new insights on dose response. PLoS ONE. 2013;8(7):e68537.

[37] Worwa G, Wheeler SS, Brault AC, Reisen WK. Comparing Competitive Fitness of West Nile Virus Strains in Avian and Mosquito Hosts. PLoS ONE. 2015;10(5):e0125668.

[38] Ziegler U, Angenvoort J, Fischer D, Fast C, Eiden M, Rodriguez AV, et al. Pathogenesis of West Nile virus lineage 1 and 2 in experimentally infected large falcons. Veterinary Microbiology. 2013;161(3):263–273.

[39] Anderson JF, Main AJ, Cheng G, Ferrandino FJ, Fikrig E. Horizontal and vertical transmission of West Nile virus genotype NY99 by *Culex salinarius* and genotypes NY99 and WN02 by *Culex tarsalis*. The American Journal of Tropical Medicine and Hygiene. 2012;86(1):134–139.

[40] Bolling BG, Olea-Popelka FJ, Eisen L, Moore CG, Blair CD. Transmission dynamics of an insect-specific flavivirus in a naturally infected *Culex pipiens* laboratory colony and effects of co-infection on vector competence for West Nile virus. Virology. 2012;427(2):90–97.

[41] Ciota AT, Chin PA, Kramer LD. The effect of hybridization of *Culex pipiens* complex mosquitoes on transmission of West Nile virus. Parasites & Vectors. 2013;6:305.

[42] Danforth ME, Reisen WK, Barker CM. Extrinsic incubation rate is not accelerated in recent California strains of West Nile virus in *Culex tarsalis* (Diptera: Culicidae). Journal of Medical Entomology. 2015;52(5):1083–1089.

[43] Dodson BL, Kramer LD, Rasgon JL. Larval nutritional stress does not affect vector competence for West Nile virus (WNV) in *Culex tarsalis*. Vector-Borne and Zoonotic Diseases. 2011;11(11):1493–1497.

[44] Dodson BL, Hughes GL, Paul O, Matacchiero AC, Kramer LD, Rasgon JL. Wolbachia enhances West Nile virus (WNV) infection in the mosquito *Culex tarsalis*. PLoS Neglected Tropical Diseases. 2014;8(7):e2965.

[45] Dohm DJ, O'Guinn ML, Turell MJ. Effect of environmental temperature on the ability of *Culex pipiens* (Diptera: Culicidae) to transmit West Nile virus. Journal of Medical Entomology. 2002;39(1):221–225.

[46] Ebel GD, Rochlin I, Longacker J, Kramer LD. *Culex restuans* (Diptera: Culicidae) relative abundance and vector competence for West Nile virus. Journal of Medical Entomology. 2005;42(5):838–843.

[47] Goddard LB, Roth AE, Reisen WK, Scott TW. Vector competence of California mosquitoes for West Nile virus. Emerging Infectious Diseases. 2002;8(12):1385–1391.

[48] Goenaga S, Kenney JL, Duggal NK, Delorey M, Ebel GD, Zhang B, et al. Potential for Co-Infection of a Mosquito-Specific Flavivirus, Nhumirim Virus, to Block West Nile Virus Transmission in Mosquitoes. Viruses. 2015;7(11):5801–5812.

[49] Hanley KA, Goddard LB, Gilmore LE, Scott TW, Speicher J, Murphy BR, et al. Infectivity of West Nile/dengue chimeric viruses for West Nile and dengue mosquito vectors. Vector-Borne and Zoonotic Diseases. 2005;5(1):1–10.

[50] Johnson B, Chambers T, Crabtree M, Arroyo J, Monath T, Miller B. Growth characteristics of the veterinary vaccine candidate ChimeriVax^{TM}-West Nile (WN) virus in *Aedes* and *Culex* mosquitoes. Medical and Veterinary Entomology. 2003;17(3):235–243.

[51] Kilpatrick AM, Meola MA, Moudy RM, Kramer LD. Temperature, viral genetics, and the transmission of West Nile virus by *Culex pipiens* mosquitoes. PLoS Pathogens. 2008;4(6):e1000092.

[52] Moudy RM, Meola MA, Morin LLL, Ebel GD, Kramer LD. A newly emergent genotype of West Nile virus is transmitted earlier and more efficiently by *Culex* mosquitoes. The American Journal of Tropical Medicine and Hygiene. 2007;77(2):365–370.

[53] Moudy RM, Zhang B, Shi PY, Kramer LD. West Nile virus envelope protein glycosylation is required for efficient viral transmission by *Culex* vectors. Virology. 2009;387(1):222–228.

[54] Reisen WK, Fang Y, Lothrop HD, Martinez VM, Wilson J, O'Connor P, et al. Overwintering of West Nile virus in southern California. Journal of Medical Entomology. 2006;43(2):344–355.

[55] Reisen WK, Fang Y, Martinez VM. Vector competence of *Culiseta incidens* and *Culex thriambus* for West Nile Virus 1. Journal of the American Mosquito Control Association. 2006;22(4):662–665.

[56] Richards SL, Mores CN, Lord CC, Tabachnick WJ. Impact of extrinsic incubation temperature and virus exposure on vector competence of *Culex pipiens quinquefasciatus* Say (Diptera: Culicidae) for West Nile virus. Vector-Borne and Zoonotic Diseases. 2007;7(4):629–636.

[57] Richards SL, Anderson SL, Lord CC. Vector competence of *Culex pipiens quinquefasciatus* (Diptera: Culicidae) for West Nile virus isolates from Florida. Tropical Medicine and International Health. 2014;19(5):610–617.

[58] Sardelis MR, Turell MJ. *Ochlerotatus j. japonicus* in Frederick County, Maryland: discovery, distribution, and vector competence for West Nile virus. Journal of the American Mosquito Control Association. 2001;17(2):137–141.

[59] Sardelis MR, Turell MJ, Dohm DJ, O'Guinn ML. Vector competence of selected North American *Culex* and *Coquillettidia* mosquitoes for West Nile virus. Emerging Infectious Diseases. 2001;7(6):1018.

[60] Tiawsirisup S, Platt KB, Evans RB, Rowley WA. A comparision of West Nile Virus transmission by *Ochlerotatus trivittatus* (COQ.), *Culex pipiens* (L.), and *Aedes albopictus* (Skuse). Vector-Borne and Zoonotic Diseases. 2005;5(1):40–47.

[61] Turell MJ, O'Guinn M, Oliver J. Potential for New York mosquitoes to transmit West Nile virus. The American Journal of Tropical Medicine and Hygiene. 2000;62(3):413–414.

[62] Turell MJ, O'Guinn ML, Dohm DJ, Jones JW. Vector competence of North American mosquitoes (Diptera: Culicidae) for West Nile virus. Journal of Medical Entomology. 2001;38(2):130–134.

[63] Vanlandingham DL, Schneider BS, Klingler K, Fair J, Beasley D, Huang J, et al. Real-Time reverse transcriptase–polymerase chain reaction quantification of West Nile virus transmitted by *Culex pipiens quinquefasciatus*. The American Journal of Tropical Medicine and Hygiene. 2004;71(1):120–123.

[64] Vanlandingham DL, McGee CE, Klinger KA, Vessey N, Fredregillo C, Higgs S. Relative susceptibilties of South Texas mosquitoes to infection with West Nile virus. The American Journal of Tropical Medicine and Hygiene. 2007;77(5):925–928.

[65] Vanlandingham DL, McGee CE, Klingler KA, Galbraith SE, Barrett AD, Higgs S. Comparison of oral infectious dose of West Nile virus isolates representing three distinct genotypes in *Culex quinquefasciatus*. The American Journal of Tropical Medicine and Hygiene. 2008;79(6):951–954.

[66] Gould DJ, Barnett HC, Suyemoto W. Transmission of Japanese encephalitis virus by Culex gelidus Theobald. Transactions of the Royal Society of Tropical Medicine and Hygiene. 1962;56(5):429–435.

[67] MACKENZIE-IMPOINVIL L, Impoinvil D, Galbraith S, Dillon R, Ranson H, Johnson N, et al. Evaluation of a temperate climate mosquito, *Ochlerotatus detritus* (= *Aedes detritus*), as a potential vector of Japanese encephalitis virus. Medical and Veterinary Entomology. 2015;29(1):1–9.

[68] Muangman D, Edelman R, Sullivan MJ, Gould DJ. Experimental transmission of Japanese encephalitis virus by Culex fuscocephala. The American Journal of Tropical Medicine and Hygiene. 1972;21(4):482–6.

[69] Van Den Hurk A, Nisbet D, Hall R, Kay B, Mackenzie J, Ritchie S. Vector competence of Australian mosquitoes (Diptera: Culicidae) for Japanese encephalitis virus. Journal of Medical Entomology. 2003;40(1):82–90.

[70] Dixon T. The distance at which sitting birds can be seen at sea. Ibis. 1977;119(3):372–375.

[71] Gibbs JP, Melvin SM. Call-response surveys for monitoring breeding waterbirds. The Journal of Wildlife Management. 1993;p. 27–34.

[72] Ronconi RA, Burger AE. Estimating seabird densities from vessel transects: distance sampling and implications for strip transects. Aquatic Biology. 2009;4(3):297–309.

[73] Pagano AM, Arnold TW. Detection probabilities for ground-based breeding waterfowl surveys. The Journal of Wildlife Management. 2009;73(3):392–398.

[74] Smith PM. Yuma Clapper Rail Study Mohave County, Arizona, 1973. State of California, The Resources Agency, Department of Fish and Game; 1974.

[75] Bart J, Stehn RA, Herrick JA, Heaslip NA, Bookhout TA, Stenzel JR. Survey methods for breeding Yellow Rails. The Journal of wildlife management. 1984;48(4):1382–1386.

[76] Piest L, Campoy J. Report of Yuma Clapper Rail Surveys at Cienega de Santa Clara, Sonora. Unpublished Report. 1998;.

[77] Kerlinger P, Sutton C. Black rail in New Jersey. Records of New Jersey Birds. 1989;15(2):22–26.

[78] Legare ML, Eddleman WR, Buckley P, Kelly C. The effectiveness of tape playback in estimating Black Rail density. The Journal of wildlife management. 1999;p. 116–125.

[79] Tecklin J. Distribution and abundance of the California black rail (*Laterallus jamaicensis coturniculus*) in the Sacramento valley region with accounts of ecology and call behavior of the subspecies. Contract Nos FG6154WM and FG6154-1WM. 1999;.

[80] Spear LB, Terrill SB, Lenihan C, Delevoryas P. Effects of Temporal and Environmental Factors on the Probability of Detecting California Black Rails. Journal of Field Ornithology. 1999;p. 465–480.

[81] Conway CJ, Gibbs J. Factors influencing detection probability and the benefits of call broadcast surveys for monitoring marsh birds. Final Report, USGS Patuxent Wildlife Research Center, Laurel, MD. 2001;.
